# TIGIT Expression on Activated NK Cells Correlates with Greater Anti-Tumor Activity but Promotes Functional Decline upon Lung Cancer Exposure: Implications for Adoptive Cell Therapy and TIGIT-Targeted Therapies

**DOI:** 10.3390/cancers15102712

**Published:** 2023-05-11

**Authors:** Md Faqrul Hasan, Tayler J. Croom-Perez, Jeremiah L. Oyer, Thomas A. Dieffenthaller, Liza D. Robles-Carrillo, Jonathan E. Eloriaga, Sanjana Kumar, Brendan W. Andersen, Alicja J. Copik

**Affiliations:** Burnett School of Biomedical Science, College of Medicine, University of Central Florida, Orlando, FL 32827, USAsanjanakumar@knights.ucf.edu (S.K.); brendanandersen@knights.ucf.edu (B.W.A.)

**Keywords:** NK cell, cancer immune cell therapy, checkpoint blockade immunotherapy, lung cancer, TIGIT blockade

## Abstract

**Simple Summary:**

Therapies targeting TIGIT have garnered tremendous interest, but have so far failed to reach primary endpoints in PhIII trials in lung cancer settings. This study examined the function of TIGIT on NK cells and how NK cells may impact the success of TIGIT therapeutics. This study demonstrated that TIGIT expression is increased on activated NK cells, including those expanded with clinical protocols for cell therapy. Activated TIGIT^+^ NK cells have a better anti-tumor response as compared to TIGIT^−^ NK cells. More importantly, higher tumor infiltration of activated NK cells correlates with better patient outcomes in lung adenocarcinoma. However, chronic TIGIT engagement with its ligands in the tumor microenvironment leads to the functional decline of NK cells, which can be prevented with anti-TIGIT. This demonstrates that TIGIT-expressing cells are not inherently exhausted but can represent NK cells with the highest anti-tumor activity. These findings support the joint application of NK cells with blocking, non-depleting anti-TIGIT for improved treatment outcomes.

**Abstract:**

Treatments targeting TIGIT have gained a lot of attention due to strong preclinical and early clinical results, particularly with anti-PD-(L)1 therapeutics. However, this combination has failed to meet progression-free survival endpoints in phase III trials. Most of our understanding of TIGIT comes from studies of T cell function. Yet, this inhibitory receptor is often upregulated to the same, or higher, extent on NK cells in cancers. Studies in murine models have demonstrated that TIGIT inhibits NK cells and promotes exhaustion, with its effects on tumor control also being dependent on NK cells. However, there are limited studies assessing the role of TIGIT on the function of human NK cells (hNK), particularly in lung cancer. Most studies used NK cell lines or tested TIGIT blockade to reactivate exhausted cells obtained from cancer patients. For therapeutic advancement, a better understanding of TIGIT in the context of activated hNK cells is crucial, which is different than exhausted NK cells, and critical in the context of adoptive NK cell therapeutics that may be combined with TIGIT blockade. In this study, the effect of TIGIT blockade on the anti-tumor activities of human ex vivo-expanded NK cells was evaluated in vitro in the context of lung cancer. TIGIT expression was higher on activated and/or expanded NK cells compared to resting NK cells. More TIGIT^+^ NK cells expressed major activating receptors and exerted anti-tumor response as compared to TIGIT^−^ cells, indicating that NK cells with greater anti-tumor function express more TIGIT. However, long-term TIGIT engagement upon exposure to PVR^+^ tumors downregulated the cytotoxic function of expanded NK cells while the inclusion of TIGIT blockade increased cytotoxicity, restored the effector functions against PVR-positive targets, and upregulated immune inflammation-related gene sets. These combined results indicate that TIGIT blockade can preserve the activation state of NK cells during exposure to PVR^+^ tumors. These results support the notion that a functional NK cell compartment is critical for anti-tumor response and anti-TIGIT/adoptive NK cell combinations have the potential to improve outcomes.

## 1. Introduction

T cell immunoreceptor with immunoglobulin and ITIM domain (TIGIT) is a major inhibitory receptor of both T cells and natural killer (NK) cells (reviewed in [1,2,3]) and is an emerging target for immune checkpoint blockade for the treatment of cancer. TIGIT competes with inhibitory receptors KIR2DL5A [4,5] and PVRIG and activating receptor CD226 (DNAM-1) to bind ligands CD155 (PVR) and CD112 (Nectin-2 or PVRL2) which are commonly expressed on cancer cells and antigen-presenting cells (APCs) (reviewed in [6]). CD96 (TACTILE) also competes for binding to PVR and has been proposed to have a function in human NK-cell-target adhesion [7], but its role as an inhibitory or activating receptor is unclear (reviewed in [8]). Additionally, PVRL3 interacts with PVR and downregulates its surface expression and availability for receptor binding [9]. TIGIT also binds PVRL4 (Nectin-4), a TIGIT-specific ligand almost exclusively expressed on tumor cells [10]. TIGIT signaling regulates the tumor immunity cycle in multiple steps [3]. It prevents T cell proliferation, cytotoxicity, and the production of proinflammatory cytokine IFNγ by these cells (reviewed in [1]). Early clinical testing of anti-TIGIT therapies has shown success. To date, 20 anti-TIGIT antibody therapies are in clinical trials for over two dozen indications, with just over a dozen more anti-TIGIT modalities in preclinical development (reviewed in [11,12,13]). However, recent phase III trials in NSCLC and ES-SCLC did not show enhancement in PFS or OS at the time of this publication [14,15]. With the mixed clinical results, there is a critical need to further understand the mechanism of function of TIGIT on immune cells to support its clinical use.

Most of the current understanding of TIGIT regulation of immune cell function has been based on T cells and/or comes from murine models, while only limited mechanistic in vitro studies of TIGIT in human NK cells exist, and these predominantly rely on NK cell lines [16,17,18,19,20,21,22], despite the same or higher TIGIT expression on NK cells compared to T cells [23,24,25]. The limited studies that examined the role of TIGIT on primary human NK cells were mostly performed with cells derived from patients with cancer or chronic infection and showed that patient NK cells express TIGIT, and, dependent on the conditions, TIGIT levels were found to be similar to those in healthy cells [22,23,26], downregulated in the case of some metastatic cancer patients [21] or upregulated in patients with chronic infection [27]. In these studies, patient’s cells were found to be dysfunctional and TIGIT blockade alone or with cytokines was shown to increase NK cell function, including IFNγ response and degranulation. While TIGIT and PD-1 combined blockade had a synergistic effect in improving tumor control and survival in mouse models and early clinical trials [12,28], in a B16 mouse model the therapeutic efficacy of PD-1 and TIGIT blockade depended on the presence of NK cells, as NK cell depletion abolished the effect [29]. These data show NK cells are an important immune population to consider in the efficacy of TIGIT blockade treatments.

Natural killer (NK) cells are first responders as part of the innate immune system, with an inherent ability to recognize and directly kill cancerous cells, and have emerged as a promising platform for next-generation of cell-based immunotherapies (reviewed in [30,31,32,33,34,35]). NK cells express a set of activating and inhibitory receptors that bind with their ligands and the balance of these inhibitory and activating receptors’ signaling determines NK cell activity [30]. NK cells not only directly kill tumor cells but also “jump-start” the anti-tumor response, as they recruit, coordinate, and activate other immune cells, including cells of the adaptive immune system through the secretion of pro-inflammatory cytokines (e.g., IFNγ and TNFα) and chemokines ([36,37,38], reviewed in [39]). Recent studies have highlighted NK cells’ critical role in the efficacy of immunotherapies where low NK cell numbers correlated with a lack of response to treatment (reviewed in [34]). The addition of adoptive NK cell treatments has the potential to improve the outcomes of current therapeutic strategies. NK cells can be efficiently expanded, viably cryopreserved without loss of efficacy [40,41,42,43,44], and are not associated with graft vs. host disease (GVHD) [45,46,47], supporting the potential safe use of cryopreserved donor-derived material as an off-the-shelf cell therapy ([48,49], reviewed in [50]). There are now large numbers of clinical trials assessing the efficacy of NK cells, including CAR-NK cells, in various cancer settings (reviewed in [51,52]).

Adoptive NK cell therapy in combination with anti-TIGIT treatment could provide an enhanced treatment option for cancer patients. In mouse models, blockade of TIGIT restored NK cell cytotoxicity, increased IFNγ and TNFα expression, and promoted tumor-specific T cell immunity [29,53,54,55]. However, there are no studies examining the effect of TIGIT expression on the cytotoxicity and function of highly activated and expanded NK cells used for adoptive cell therapy. Here, a novel exhaustion model was utilized in combination with kinetic live-cell imaging assays and sequencing to assess the impact of long-term exposure to lung cancer spheroids on the function of NK cells expanded with PM21 particles (PM21-NK cells) [48,49,56]. PM21-NK cells are clinically utilized in currently ongoing trials for cancer treatment (NCT04220684 and NCT05115630). Ex vivo expansion and/or activation upregulated TIGIT on NK cells. TIGIT-expressing cells expressed more activating receptors and had a higher frequency of cells producing effector cytokines and degranulating in response to target cell engagement. LUAD-TCGA database analysis demonstrated that TIGIT expression in the tumor tissue positively correlated with activating NK cells, while the presence of activating NK cells correlated with better outcomes. Expanded NK cells were found to be more resistant to TIGIT inhibition, where TIGIT blockade did not increase the anti-tumor activities of PM21-NK cells against K562 cells or lung cancer cells in monolayers, or within the first 24–32 h of spheroid exposure. However long-term spheroid exposure resulted in decreased cytotoxicity and function that could be preserved by the inclusion of TIGIT blockade. TIGIT blockade increased PM21-NK cell cytotoxicity against 3D lung cancer spheroids and prevented PVR-mediated dysfunction after exposure to tumor spheroids. TIGIT blockade upregulated multiple gene sets related to NK cell anti-tumor responses, including inflammatory response-related genes, TNFα signaling via NFкB, and IFNγ response-related gene sets. The implications of these findings on understanding TIGIT-targeted therapies and the use of adoptive NK cell/anti-TIGIT combinations are discussed.

## 2. Materials and Methods

### 2.1. Cell Culture

Buffy coats (leukocyte source) from de-identified healthy donors were used as a source of NK cells and were purchased from a local blood bank (OneBlood). Peripheral blood mononuclear cells (PBMC) were separated by density gradient (Ficoll-Paque Plus solution; GE Healthcare, Chicago, IL, USA) and cryopreserved for further use. NK cells were expanded with PM21 particles as described previously [48,49,56]. Briefly, T-cell-depleted PBMCs (EasySep CD3-positive selection kit; StemCell Technologies, Vancouver, Canada) were stimulated with 200 μg/mL PM21 particles and cultured for 2–3 weeks in SCGM media (CellGenix GmbH, Freiburg im Breisgau, Germany) and RPMI media with 100 U/mL IL-2 (PeproTech, Cranbury, NJ, USA). For activated NK cells, T-cell-depleted PBMCs were stimulated overnight with IL-2 (1000 U/mL) or IL-12 (10 µg/mL) + IL-15 (100 µg/mL) + IL-18 (50 µg/mL). Cancer cell line K562, A549, NCI-H358, and NCI-H1975 cells (ATCC) and NCI-H1299 (a generous gift from Dr. Griffith Parks, UCF) were maintained in RPMI media with 10% FBS, 1% antibiotic/antimycotic, and 2 mM Glutamax. A549-NLR, NCI-H358-NLR, NCI-H1975-NLR, and NCI-H1299-NLR cells were generated through stable transduction using commercial Nuclight Red Lentivirus (Sartorius). All cell lines were positively selected via puromycin selection followed by sorting on uniform positive populations (BD FACS Aria II). All cells were maintained in a humidified atmosphere at 37 °C supplemented with 5% (*vol*/*vol*) CO_2_ in air. Cell lines were routinely assessed for mycoplasma (E-Myco Plus Mycoplasma PCR Detection Kit, Bulldog-Bio, Inc., Portsmouth, NH, USA) and authenticated via human STR profiling (serviced by ATCC).

### 2.2. Stable Cell Line Generation

PVR-expressing K562-GFPLuc cells were generated via stable transduction using lentiviral particles generated in-house (VectorBuilder Inc., Chicago, IL, USA) containing PVR coding gene sequences and sorted for positive and negative populations with a BD FACSAria II Cell Sorter (BD Biosciences, Franklin Lakes, NJ, USA). PVR^+^ and PVR^−^-K562-GFPLuc cell lines were cryopreserved until needed.

### 2.3. qRT-PCR

NK cells were selected (EasySep CD56-positive selection kit StemCell Technologies, Vancouver, BC, Canada) before and after expansion, and total RNA was isolated (Direct-zol RNA Microprep kit; Zymo Research, Irvine, CA, USA). cDNA was synthesized (High-Capacity cDNA Reverse Transcription Kit; Applied Biosystems, Waltham, MA, USA) and a gene expression primer set for TIGIT (QuantiTect Primer Assay; Qiagen, Hilden, Germany) was used to determine RNA expression levels by qRT-PCR (Quantstudio 7 PCR system, Applied Biosystems, USA). EIF3D and RPL13A were used as control genes. The 2^−ΔΔCT^ method [57] was used to determine the relative RNA expression of the target gene.

### 2.4. Flow Cytometry

The following antibodies were used for flow cytometry analysis: CD56-PE (clone:5.1H11), CD56-APC/Fire™750 (clone:NCAM), CD56-AF^®^647 (clone:5.1H11), CD3-FITC (Clone:UCHT1), TIGIT-PE/Cy7 (Clone:A15153G), CD96-PE (Clone:NK92.39), DNAM-1-FITC (Clone:TX25), PVRIG-APC (Clone:W16216D), PVR-PE (Clone:SKIL2), PVRL2-APC (Clone:TX31), PVRL4-AF488 (Clone:337516), NKp30-PE (Clone:P30-15), NKp46-PE/Dazzle™594 (Clone:9E2), CD16-PeCy5 (Clone:3G8), NKG2D-APC (Clone:1D11), NKp44-PE-Cy7 (Clone:P44-8), LAG-3-FITC (Clone:11C3C65), PD-1-PE-Dazzle™594 (Clone:EH12.2H7), TIM-3-PE (Clone:F38-2F2), TNFα-PE/Dazzle™594 (Clone:MAB11), IFNγ-PerCP5.5 (Clone:B27), CD107a-PE (Clone:H4A3), CD3-PerCP-eF710 (Clone:OKT3), and NKG2A-APC (Clone:Z199). NK cells were stained with pre-conjugated protein-specific or the corresponding isotype control antibodies. All samples were acquired on a Cytoflex (Beckman Coulter, Brea, CA, USA) or Northern Lights 2000 Full Spectrum (Cytek, Fremont, CA, USA) flow cytometer and analyzed with FlowJo software (v10.6.2). An example gating strategy for NK cells is shown in Supplementary Materials Figure S1.

### 2.5. Kinetic Live-Cell Imaging Cytotoxicity Assays

Kinetic live-cell imaging cytotoxicity assays were performed as previously described [58]. Lung cancer cell lines A549-NLR, NCI-H358-NLR, NCI-H1975-NLR, and NCI-H1299-NLR, stably expressing nuclear red fluorescent protein (Nuclight Red; NLR) for tracking were used as target cells. For monolayer cytotoxicity assays, 6000 cancer cells were seeded per well in a flat-bottom 96-well plate the day prior to adding NK cells. For spheroid cytotoxicity assays, 5000 cancer cells were seeded in a 96-well clear round-bottom ultra-low attachment microplate (Corning, Corning, NY, USA), centrifuged at 130× *g* for 10 min, and incubated for 3 days to form spheroids. Cancer cell monolayers or spheroids were then co-cultured with NK cells at the indicated effector-to-target (E:T) ratios in the presence of Ultra-LEAF isotype or anti-TIGIT antibodies (Biolegend, San Diego, CA, USA). Monolayers were imaged for 72 h, while spheroid experiments were conducted for 7 days with an IncuCyte^®^ S3 Live-Cell Analysis System (Sartorius, Göttingen, Germany). Target tumor cell growth was tracked over time by red object count per well (ROC) in 2D assays and total red object integrated intensity (ROII) (RCU × μm^2^/Image) in 3D assays. Relative growth of the target cells alone or in the presence of NK cells with or without TIGIT blockade was determined by normalizing ROC or ROII to the value at time 0 (ROC_t_/ROC_t=0_ or ROII_t_/ROII_t=0_) when NK cells were initially added to determine normalized ROC (nROC) or normalized ROII (nROII) [59]. Cytotoxicity (%) was then determined based on the following equations:

$${2DCytotoxicity}^{E:T}\left(\%\right)=(1-(\frac{{nROC}^{E:T}}{{nROC}^{T}}))\times 100$$

$${3DCytotoxicity}^{E:T}\left(\%\right)=\left(1-\left(\frac{{nROII}^{E:T}}{{nROII}^{T}}\right)\right)\times 100$$

### 2.6. In Vitro Exhaustion Model

A549-NLR cells (5000/well) were seeded in a 96-well clear round-bottom ultra-low attachment microplate (Corning, Corning, NY, USA), centrifuged at 130× *g* for 10 min, and incubated for 3–4 days to form spheroids. NK cells were then added in the presence of Ultra-LEAF isotype or anti-TIGIT antibodies (Biolegend, San Diego, CA, USA). After 7 days of incubation, NK cells were stimulated with PVR^−^ or PVR^+^-K562-GFPLuc cells for 4–6 h in the presence of Brefeldin A (eBioscience, San Diego, CA, USA) and Golgi Stop™ (BD Biosciences, Franklin Lakes, NJ, USA). Samples were harvested and stained with CD56, CD3, and CD107a antibodies, fixed and permeabilized (eBioscience IC Fixation and Permeabilization buffers), and probed with antibodies for IFNγ and TNFα, followed by analysis using flow cytometry.

### 2.7. RNA-Seq

NK cells were set up as described in the exhaustion model. After 7 days of coincubation, NK cells were isolated with an NK cell selection kit (EasySep CD56+ selection kit; StemCell technologies, Vancouver, BC, Canada) and analyzed by flow cytometry (Northern Lights 2000 Full Spectrum, Cytek, Fremont, CA, USA) to determine NK cell purity. Total RNA was extracted from the NK cells (Direct-zol Microprep kit, Zymo research, Irvine, CA, USA). RNA quality (RIN value) was determined by TapeStation and used for polyA selection, library preparation, and RNA sequencing (Genewiz, Inc., South Plainfield, NJ, USA). Raw RNA-seq data (Fastq) were analyzed with FastQC for quality control [60]. Trimmomatic was used for trimming adaptor and low-quality reads [61], HISAT2 for mapping genes with the hg38 human genome, and Stringtie for assembly and quantification of read counts [62]. Combat-seq was used to remove batch effects among samples [63] and EdgeR to normalize gene expression and determine differentially expressed genes [64]. A ranked gene list was generated by multiplying log_2_-fold change and –log_10_ (*p*-value) of individual genes obtained from EdgeR and used for pre-ranked gene set enrichment (GSEA) analysis to determine enriched hallmark-gene sets [65].

### 2.8. TIMER2.0-Based Analyses

The TIMER2.0 webserver [66,67,68] was used to report NK cell immune infiltration, gene expression, and patient survival correlations from the lung adenocarcinoma patient cohort of The Cancer Genome Atlas Program (TCGA) (*n* = 515). Correlation between TIGIT and NK cell infiltration was analyzed after tumor purity adjustment (partial Spearman’s correlation) using the CIBERSORT immune deconvolution method in absolute mode. The statistical significance of differential expression between tumor and adjacent normal tissues was analyzed by the Wilcoxon test. The Cox proportional hazard model was used to determine the survival of lung adenocarcinoma patients using the upper and lower 25% of patients. The hazard ratio (HR) and *p*-value for Kaplan–Meier curves were determined to understand clinical relevance.

### 2.9. Statistics

Statistical analysis was performed by GraphPad Prism 9.3.1. Paired or unpaired two-tailed Student’s *t*-tests were used unless noted in the figure legend. All experiments were performed for at least 3 biological replicates. *p*-values less than 0.05 were considered statistically significant. *p*-values are shown as * if *p* < 0.05, ** if *p* < 0.01, *** if *p* < 0.001, and **** if *p* < 0.0001.

## 3. Results

### 3.1. PM21-Particle-Expanded or Cytokine-Activated NK Cells Have Increased TIGIT Expression

**Figure 1:**
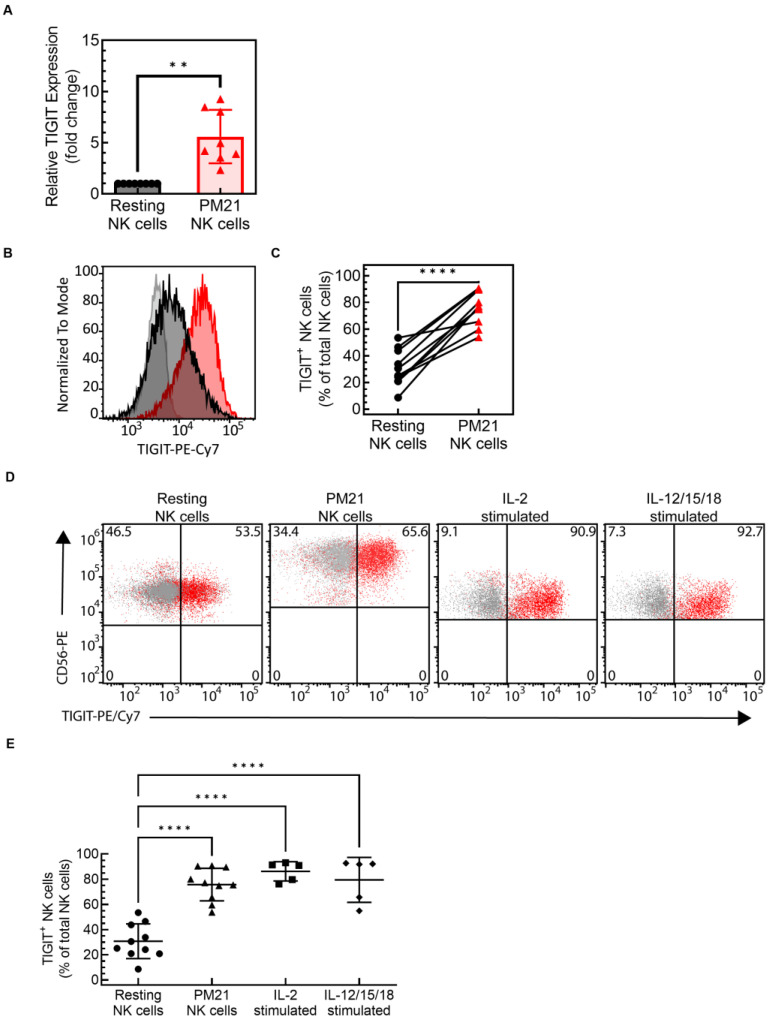
Cytokine-activated or PM21-particle-expanded NK cells highly expressed TIGIT. NK cells were either expanded with PM21 particles from T-cell-depleted PBMCs (PM21-NK cells) or were isolated from PBMCs by negative selection and analyzed for TIGIT expression directly (resting NK cells) or after overnight stimulation with cytokines. TIGIT expression was analyzed on the mRNA level by qPCR and on the protein level by flow cytometry. (**A**) TIGIT RNA level was on average 5.6 ± 2.6-fold greater in PM21-particle-expanded NK cells compared to resting NK cells (*N* = 8 donors). TIGIT expression was measured on the protein level by flow cytometry. (**B**) A representative histogram overlay is shown comparing PM21-NK cells (red) and resting NK cells (black) to isotype control (gray). (**C**) Summary data from 10 donors showed TIGIT protein expression significantly increased in PM21-NK cells (red triangles) compared to donor-matched resting NK cells (black circles). (**D**) Flow cytometry was also used to analyze TIGIT expression on NK cells activated by other methods, with dot plots showing examples of TIGIT expression on resting and activated cells as indicated above the graphs (red—anti-TIGIT vs. black—isotype ctrl.). (**E**) Consistent with PM21-NK cells (triangles), TIGIT expression is also increased in NK cells activated overnight with 1000 U/mL of IL-2 (squares) or 10 µg/mL IL-12, 100 µg/mL IL-15, and 50 µg/mL IL-18 (diamonds) compared to resting NK cells (circles) (*N* = 5–10 donors). Data are presented as a mean with error bars representing standard deviation (SD), scatter plots with donor-pair lines, or scatter plots with mean and SD. Paired two-tailed Student’s t-tests were used to compare TIGIT expression in PM21-NK cells to that in resting cells and one-way ANOVA corrected for multiple comparisons using a Turkey post hoc test was used to compare TIGIT expression across different NK cell activation methods using GraphPad Prism software v. 9.3.1. *p*-values are shown as ** if *p* < 0.01, and **** if *p* < 0.0001.

NK cells obtained from healthy donors were expanded using PM21 particles [48,49]. This feeder cell-free expansion technology utilizes plasma membrane particles derived from K562-mbIL21-41BBL cells (PM21 particles) to stimulate NK cell proliferation, resulting in an average 1700-fold expansion of NK cells in 2 weeks (*N* = 113 from 18 donors, Supplementary Materials Figure S2). Resting NK cells isolated from PBMCs, and NK cells expanded from matching donors using PM21 particles (PM21-NK cells) were analyzed for TIGIT expression by qRT-PCR and flow cytometry. TIGIT was upregulated on the RNA level in PM21-NK cells compared to resting NK cells, ranging from 2.3-fold to 9.3-fold (average 5.6 ± 2.6-fold; *N* = 8, *p* < 0.002) (Figure 1A). Furthermore, the percentage of TIGIT^+^ NK cells was increased in PM21-NK cells compared to resting NK cells (Figure 1B), with the expression ranging from 9% to 54% in resting NK cells and 54% to 90% in PM21-NK cells (*N* = 9, *p* < 0.0001) (Figure 1C). To determine if other NK cell activation methods also increase TIGIT expression, T-cell-depleted PBMCs were stimulated overnight with either IL-2 (1000 U/mL) or the combination of IL-12 (10 µg/mL), IL-15 (100 µg/mL), and IL-18 (50 µg/mL), and the percentage of NK cells expressing TIGIT was compared to resting NK cells (Figure 1D). TIGIT expression was upregulated on IL-2-activated NK cells (86 ± 7%, *p* < 0.0001) and IL-12/15/18-activated NK cells (79 ± 18%, *p* < 0.0001) compared to resting NK cells (29 ± 13%), and was comparable to the level of expression on PM21-NK cells (74 ± 13%) (Figure 1E). Altogether, these findings demonstrated that NK cells, activated with either PM21 particles or cytokines, upregulated TIGIT on both the RNA and surface protein levels.

### 3.2. More TIGIT^+^ PM21-NK Cells Expressed Activating and Inhibitory Receptors Compared to TIGIT^−^ PM21-NK Cells, and upon Stimulation with K562 Cells, More TIGIT^+^ NK Cells Produced Effector Cytokines and Had Surface CD107a

**Figure 2:**
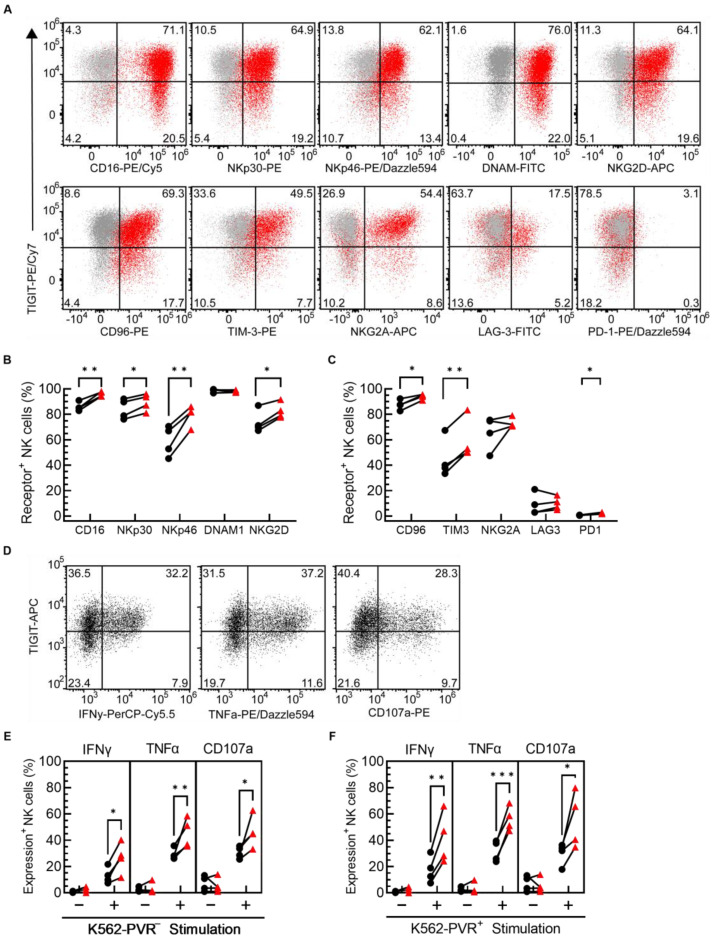
TIGIT^+^ NK cells have increased expression of NK cell receptors compared to TIGIT^−^ NK cells and have enhanced anti-tumor function. NK cells from four donors were expanded with PM21 particles from T-cell-depleted PBMCs for 12 days. Expression of NK cell activating and inhibitory receptors was determined by flow cytometry and gated on TIGIT^−^ or TIGIT^+^ NK cells. (**A**) Representative flow cytometry dot plots with gating are shown comparing NK cells with isotype control (gray) or the indicated receptor-specific antibody (red). The percentages of NK cells expressing (**B**) activating receptors CD16, NKp30, NKp46, DNAM1, and NKG2D and (**C**) inhibitory receptors CD96, TIM3, NKG2A, LAG3, and PD1 were determined for TIGIT^+^ NK cells (red triangles) and TIGIT^−^ NK cells (black circles) (*N* = 4 donors, avg. of duplicates). PM21-NK cells were stimulated with either PVR^−^ or PVR^+^-K562 cells and the percentage of TIGIT^−^ or TIGIT^+^ NK cells expressing IFNγ, TNFα, or CD107a was determined by flow cytometry with (**D**) representative dot plots shown for stimulation with PVR^−^ for each effector function measured. Significantly more TIGIT^+^-PM21-NK cells (red triangles) produced IFNγ and TNFα, and expressed CD107a after stimulation with either PVR^−^ (**E**) or PVR^+^ (**F**) K562 cells compared to TIGIT^−^-PM21-NK cells (black circles) (*N* = 4 donors, avg. of 2–3 replicates). Data are presented as scatter plots with donor-pair lines. Statistical significance was determined by multiple paired *t*-tests. *p*-values are shown as * if *p* < 0.05, ** if *p* < 0.01 and *** if *p* < 0.001.

To determine if the expression of TIGIT is associated with any changes to the phenotype and/or activation state of NK cells, the level of expression of major activating and inhibitory receptors was compared between TIGIT^+^ and TIGIT^−^ PM21-NK cells, determined by flow cytometry gating (Figure 2A, Supplementary Figure S1). Differences were evaluated on cells prior to day 14 of expansion when the expression is not at the maximal level and differential expression can still be assessed. In general, expression of activating and other inhibitory receptors was increased on TIGIT^+^ PM21-NK cells compared to TIGIT^−^ PM21-NK cells, as summarized in Figure 2B,C. The percentage of NK cells expressing activating receptors CD16, NKp30, NKp46, DNAM-1, and NKG2D varied between donors but was increased (*p* = 0.02 or less) for TIGIT^+^ vs. TIGIT^−^ NK cells in donor-matched pairs for all activating receptors, except for DNAM-1. DNAM-1 was ubiquitously and highly expressed on all PM21-NK cells, averaging more than 98% of both TIGIT^−^ and TIGIT^+^ NK cells (Figure 2B). The inhibitory receptors CD96, TIM-3, NKG2A, and LAG-3 were also expressed on PM21-NK cells, while PD-1 was detected on fewer than 2% of PM21-NK cells (Figure 2C). A significantly higher percentage of TIGIT^+^ NK cells expressed CD96 (*p* = 0.01) and TIM-3 (*p* = 0.008) compared to donor-matched TIGIT^−^ NK cells, while expression of NKG2A and LAG-3 were not significantly different. These findings showed that TIGIT^+^ NK cells expressed higher levels of important activating and some inhibitory receptors typically induced upon activation.

Next, the functional status of TIGIT^+^ vs. TIGIT^−^ PM21-NK cells was assessed to determine if TIGIT expression on NK cells also corresponds to higher function. For this, the production of effector cytokines and degranulation in unstimulated PM21-NK cells or cells stimulated with either PVR^−^ or PVR^+^-K562 cells was measured and compared based on TIGIT expression (Figure 2D). Significantly more TIGIT^+^ PM21-NK cells than TIGIT^−^ cells produced IFNγ (27 ± 12% vs. 13 ± 6%, *p* = 0.014) and TNFα (45 ± 11% vs. 30 ± 4%, *p* = 0.005) and stained for more surface CD107a (46 ± 12% vs. 31 ± 5%, *p* = 0.013) in response to PVR^−^-K562 cells (Figure 2E). Similarly, PVR^+^-K562 cell stimulation resulted in more TIGIT^+^ PM21-NK cells than TIGIT^−^ cells producing IFNγ (41 ± 19% vs. 17 ± 10%, *p* = 0.009) and TNFα (56 ± 9% vs. 32 ± 8%, *p* < 0.001) and with surface CD107a (55 ± 21% vs. 30 ± 8%, *p* = 0.013) (Figure 2F). There was no inhibition of TIGIT^+^ PM21-NK cells stimulated with PVR^+^-K562 cells compared to PVR^−^; in fact, the percentage of IFNγ^+^ and TNFα^+^ NK cells trended higher for PVR^+^-K562 stimulation (IFNγ 41 ± 19% vs. 27 ± 12%, *p* = 0.06; TNFα 56 ± 9% vs. 45 ± 11%, *p* = 0.07). This suggests that the response to PVR is overpowered by signaling through activating receptors DNAM-1 and/or NKG2D which are highly expressed on PM21-NK cells, while TIGIT inhibition is less evident. Consistent with the observations made with regard to PM21-NK cells, when NK cells were activated with IL-2 or IL-12/15/18, more TIGIT^+^ NK cells than TIGIT^−^ cells produced TNFα and had more cells with surface CD107a in response to both PVR^−^ and PVR^+^ K562 cell stimulation (Supplementary Materials Figure S3). However, no significant difference was observed between TIGIT^+^ and TIGIT^−^ NK cells in IFNγ, with overall low levels of IFNγ-expressing cells with O/N IL-2 activation (less than 14%) and overall high levels of IFNγ-expressing cells even without any stimulation with O/N IL-12/15/18 (more than 40%). The high basal level of IFNγ secretion with O/N IL-12/15/18 could be overpowering the response, not allowing for the detection of induction by target cell stimulation. No significant difference in effector cytokine production or degranulation between TIGIT^+^ and TIGIT^−^ PM21-NK cells was observed in response to cytokine stimulation with IL-12/15/18 (Supplementary Materials Figure S4), indicating this greater function is specific to anti-tumor response. Taken together, these data suggest that TIGIT^+^ NK cells represent a more activated cell subpopulation of NK cells with greater anti-tumor response capacity, and those cells are not inherently exhausted.

### 3.3. TIGIT Blockade Enhanced PM21-NK Cell Cytotoxicity against 3D Lung Tumor Spheroids

**Figure 3:**
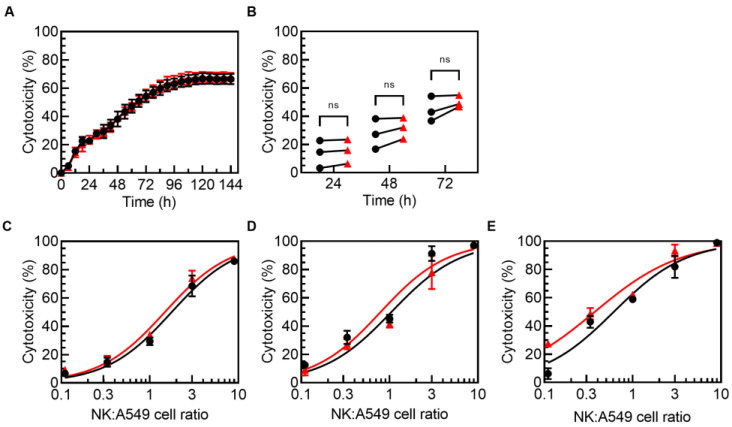
TIGIT blockade did not enhance PM21-NK cell cytotoxicity against A549 lung tumor monolayers. NK cells were expanded with PM21 particles from T-cell-depleted PBMCs obtained from multiple donors for 14–16 days. Cytotoxicity against A549-NLR lung cancer cells in a monolayer, measured by kinetic live-cell imaging, was not significantly improved in the presence of anti-TIGIT antibodies compared to isotype control. (**A**) Representative cytotoxicity time courses from one donor are shown in the presence of 0.3:1 NK cells:A549 cells either with isotype control (black circles) or in the presence of anti-TIGIT antibodies (red triangles). (**B**) Summary plot comparing cytotoxicity at 24, 48, and 72 h for NK cells from multiple donors at 0.3:1 of NK:A549 cells is shown (*N* = 3). Concentration-dependent cytotoxicity curves are shown for one donor at multiple NK cells:A549 cell ratios at 24 h (**C**), 48 h (**D**), and 72 h (**E**). Statistical significance was determined by multiple paired *t*-tests and represented as ns if *p* > 0.05.

To access the effect of TIGIT blockade on PM21-NK cell anti-tumor functions, PM21-NK cell cytotoxicity against A549 lung tumor cells was examined. This cell line expresses TIGIT ligands PVR and PVRL2 but not PVRL3 or PVRL4 (Table 1, Supplementary Figure S5). PM21-NK cells were co-cultured with 2D A549 lung cancer cell monolayers at a 0.33:1 NK:A549 cell ratio in the presence of anti-TIGIT antibodies or isotype controls and cytotoxicity was measured with a live-cell imaging assay. No significant enhancement of PM21-NK cell killing occurred with TIGIT blockade over 72 h (cytotoxicity in the presence of isotype control vs. anti-TIGIT antibodies was 14 ± 10% vs. 15 ± 9% at 24 h, 27 ± 11% vs. 31 ± 7% at 48 h, and 44 ± 9% vs. 50 ± 4% at 72 h, *N* = 3 donors) (Figure 3A,B). Concentration-dependent cytotoxicity curves were determined for each timepoint for one donor and no difference in killing was observed with or without TIGIT blockade (area under the curve was 559 ± 18% × cell ratio vs. 525 ± 24% × cell ratio at 24 h, 689 ± 17% × cell ratio vs. 629 ± 37% × cell ratio at 48 h, and 751 ± 14% × cell ratio vs. 697 ± 25% × cell ratio at 72 h) (Figure 3C–E).

**Figure 4:**
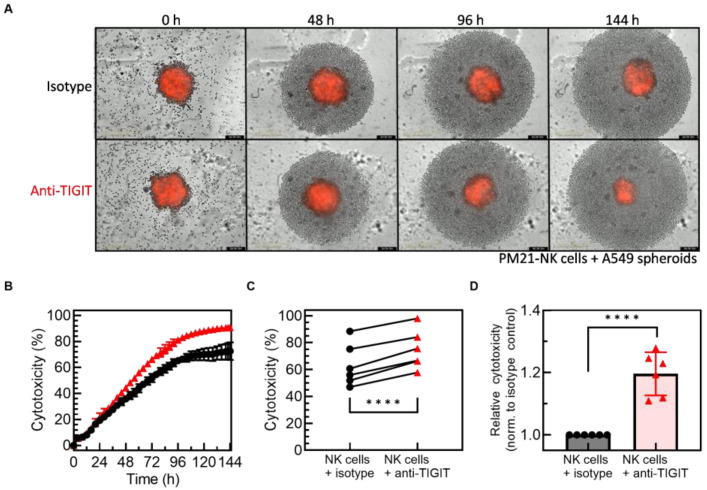
TIGIT blockade enhanced PM21-NK cell cytotoxicity against A549 lung tumor spheroids. Expanded NK cells were co-cultured with A549 tumor spheroids for 7 days. NK cell cytotoxicity was determined by kinetic live-cell imaging. Representative images at 10× magnification from the live-cell imaging cytotoxicity assay of NLR-expressing A549 cancer cell spheroids incubated with 10,000 NK cells in the presence of anti-TIGIT or isotype control after 0, 48, 96, and 144 h showed increased NK cell cytotoxicity in the presence of anti-TIGIT antibodies against A549 spheroids (**A**). Representative cytotoxicity time courses from one donor are shown with isotype control (black circles) or anti-TIGIT (red triangles) antibodies present (**B**). Summary plot comparing NK cell cytotoxicity from multiple donors at 72 h at 1:1 NK:A549 ratio shows that TIGIT blockade significantly increased cytotoxicity (*N* = 6 donors, avg. of 2–3 replicates) (**C**), and relative cytotoxicity increased for each donor when normalized to isotype control (**D**). Data are presented as scatter plots with donor-pair lines or as mean with error bars representing standard deviation. Statistical significance was determined by multiple paired *t*-tests. For concentration-dependent cytotoxicity curves, the area under the curve (AUC) was determined and compared by unpaired *t*-tests. *p*-values are shown as **** if *p* < 0.0001.

To determine if blockade of TIGIT affects PM21-NK cell cytotoxicity against lung cancer spheroids that better mimic the cancer environment, PM21-NK cells were co-cultured with A549 cell spheroids, and their killing over time as well as NK cell phenotype at the end of the co-culture were assessed. PM21-NK cells were exposed to tumor spheroid for 7 days in the presence of anti-TIGIT or isotype control antibodies. Representative videos and images at several time points show the increased killing of A549 spheroids in the presence of anti-TIGIT after 48 h (Supplementary Figure S6A, Figure 4A), apparent by the smaller size of resulting spheroids (Figure 4A) and reduced relative spheroid growth (Supplementary Figure S6B). Cytotoxicity curves over time were determined and representative curves from one donor and summary cytotoxicity data from multiple donors are shown in Figure 4B–D. The cytotoxicity was increasing over time with a comparable rate of killing observed between the control and anti-TIGIT samples during the first 48 h (Supplementary Figure S6A). Killing slowed down after 28 h in the absence of anti-TIGIT, while the killing rate remained unchanged when NK cells were with anti-TIGIT until 72 h, resulting in greater killing observed in the presence of anti-TIGIT over time. TIGIT blockade enhanced PM21-NK cell cytotoxicity against A549 spheroids at 72 h by an average of 20% across donors (*p* < 0.0001, 1:1 PM21-NK cells:A549 cells, *N* = 6) (Figure 4C,D). Expression of multiple activating and inhibitory receptors was evaluated on unexposed control PM21-NK cells and PM21-NK cells from three donors after exposure to A549 spheroid in the presence of isotype control or anti-TIGIT antibodies. Tumor exposure alone or with TIGIT blockade did not change the frequency of NK cells expressing inhibitory receptors TIM-3, NKG2A, LAG-3, PD-1, or CD96 compared to isotype control or unexposed NK cells (Supplementary Figure S7). Similarly, there was no difference in the expression of activating receptors CD16, NKG2D, NKp30, NKp46, and DNAM-1 after tumor exposure with or without TIGIT blockade, although there was a trend toward a decreased expression of NKG2D upon spheroid exposure, particularly with isotype control (Supplementary Figure S7).

To confirm that the enhancement in NK cell cytotoxicity against lung cancer spheroids upon TIGIT blockade is not limited to a single cell line, testing of the anti-TIGIT antibody was also performed in the NCI-H1299, NCI-H358, and NCI-1975 lung cancer cell lines. These cell lines also highly express PVR and PVRL2 while H358 additionally expresses PVRL4 among the TIGIT ligands assessed (Table 1).

**Figure 5:**
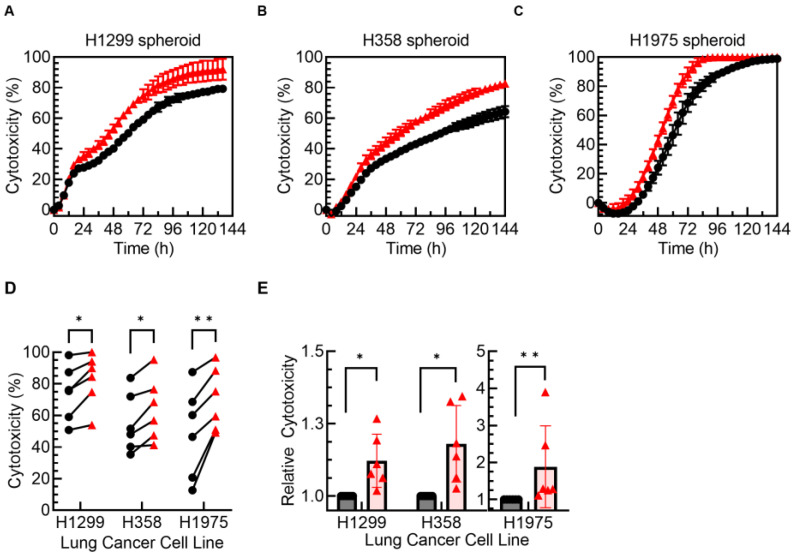
TIGIT blockade enhanced NK-cell-mediated killing in multiple 3D lung tumor spheroid models. NK cells were expanded with PM21 particles from T-cell-depleted PBMCs obtained from multiple donors for 14–16 days. Expanded NK cells were co-cultured with NCI-H1299-NLR, NCI-H358-NLR, or NCI-1975-NLR lung tumor spheroids for 7 days. NK cell cytotoxicity time courses were determined by kinetic live-cell imaging. Representative cytotoxicity curves from one donor are shown against H1299 (**A**), H358 (**B**), and H1975 (**C**) spheroids either with isotype control (black circles) or TIGIT (red triangles) antibodies present. Summary plot comparing NK cell cytotoxicity from multiple donors at 72 h shows that TIGIT blockade significantly increased cytotoxicity (**D**) and enhanced relative cytotoxicity when cytotoxicity with anti-TIGIT for each donor was normalized to isotype control (**E**) (E:T ratios used were 3:1 for NCI-H1299, 1:1 for NCI-H358, and 1:3 for NCI-H1975; *N* = 6 donors, avg. of 3 replicates). Data are presented as scatter plots with donor-pair lines or as mean with error bars representing standard deviation. Statistical significance was determined by multiple paired *t*-tests. *p*-values are shown as * if *p* < 0.05 and ** if *p* < 0.01.

PM21-NK cell cytotoxicity against spheroids of these cell lines was determined using the live-cell imaging assay. Each cell line was killed at different rates, but all demonstrated enhanced cytotoxicity upon TIGIT blockade after the 24 h time point, with the effect increasing over time (Figure 5A–C). While there was donor-dependent variability in the extent of killing, TIGIT blockade increased cytotoxicity in donor-matched comparisons against all cell lines tested (NCI-H1299 *p* = 0.02, NCI-H358 *p* = 0.01, and NCI-1975; *p* = 0.005) and modestly enhanced the killing of H1299 (on average by 12%) and H358 (by 18%), while strongly enhancing killing of H1975 (by 88%) (Figure 5D,E). Collectively, these findings indicate that in 3D spheroid models, TIGIT blockade enhanced the cytotoxicity of PM21-NK cells over time against lung cancer cells without a change in the phenotype of the NK cells. The enhancement difference was not observed during the first 24–36 h but increased over time, with the largest differential observed after 72 h.

### 3.4. TIGIT Blockade Preserved PM21-NK Cell Effector Function against PVR-Positive Cancer Cells after Co-Culture with Cancer Cell Spheroids

**Figure 6:**
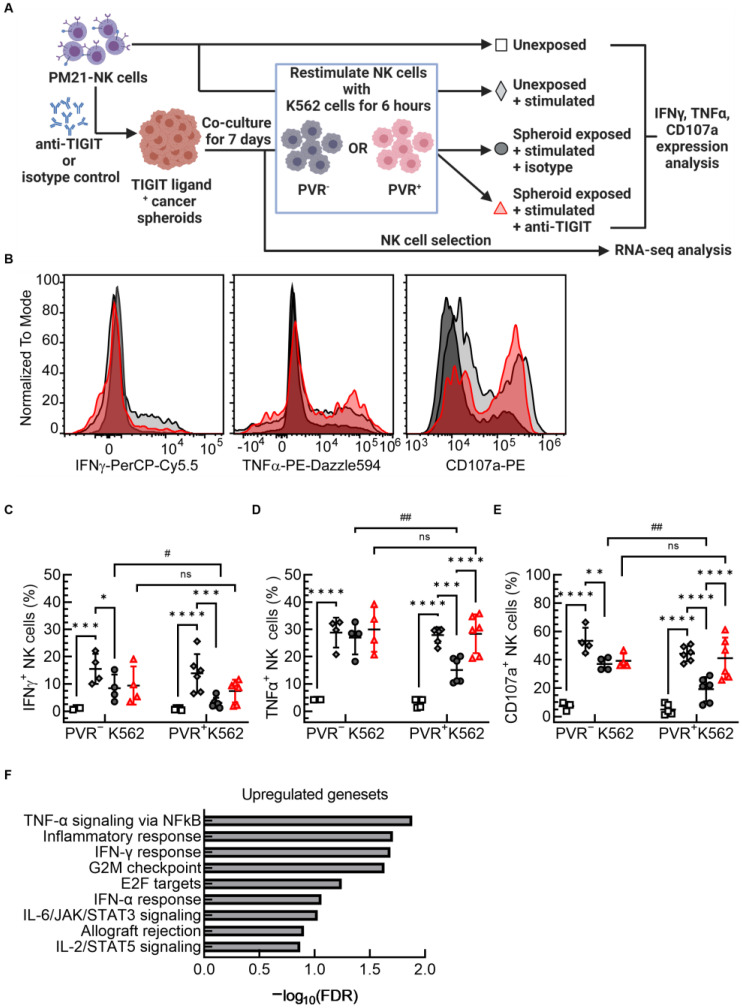
TIGIT blockade prevents PVR-mediated NK cell exhaustion during spheroid exposure. NK cells were expanded from T-cell-depleted PBMCs for 14–16 days. Expanded NK cells were co-cultured with A549 spheroids for 7 days in the presence of anti-TIGIT or isotype control. After 7 days of co-culture, NK cells were stimulated with K562 cancer cells with or without PVR expression for 4–6 h in the presence of Brefeldin A and Golgi Stop, and NK cell expression of surface CD107a, IFNγ, and TNFα was analyzed with flow cytometry. Unexposed PM21-NK cells, either unstimulated or stimulated, were used as controls. Additionally, NK cells were selected after co-culture with A549 spheroids and used for RNA extraction and transcriptomic analysis. A schematic of the experiment is shown in (**A**). IFNγ, TNFα, and CD107a expression on NK cells was determined by flow cytometry, and representative histograms are shown overlaying unexposed stimulated PM21-NK cells (light gray fill with black outline) with PM21-NK cells that were tumor exposed in the presence of a blocking antibody isotype control (dark gray fill with black outline) or anti-TIGIT (red fill and outline) and stimulated with PVR^+^ K652 cells for all shown conditions (**B**). Unstimulated, unexposed NK cells (open squares), unexposed stimulated NK cells (gray filled diamonds), NK cells that were tumor-exposed in the presence of isotype (dark gray filled circles) or anti-TIGIT antibodies (red filled triangles) are shown for either re-stimulation with PVR^−^-K562 cells or PVR^+^-K562 cells. TIGIT blockade preserved IFNγ (**C**), TNFα (**D**), and CD107a (**E**) expression to levels of unexposed NK cells, when re-challenged with PVR^+^-K562 cells (*N* = 4–6 donors, avg. of 2 replicates). GSEA analysis of RNA-seq data from 3 donors shows that TIGIT blockade upregulated IFNγ, TNFα, and other related inflammation response gene sets. Summary graphs show upregulated gene sets upon TIGIT blockade based on their −log_10_(FDR) (**F**). Data are presented as scatter plots or bar graphs with error bars representing standard deviation. Statistical significance was determined by 2-way ANOVA with *p*-values shown as * if *p* < 0.05, ** if *p* < 0.01, *** if *p* < 0.001, and **** if *p* < 0.0001 for comparing each exposure and stimulation condition. Statistical significance was determined by unpaired *t*-tests with *p*-values shown as ns *p* > 0.05, # if *p* < 0.05, or ## *p* < 0.01.

NK cell exhaustion can occur in the context of the tumor microenvironment whereby long-term exposure to tumors leads to decreased effector function, altered phenotype, or decreased killing. The mechanisms leading to NK cell exhaustion are not well defined, although recent studies have shown that exacerbated inhibitory receptor signaling plays a role (reviewed in [69]). Previous studies have reported that chronic inhibitory signaling promotes the exhaustion of cytotoxic immune cells [69] and TIGIT has been associated with NK cell exhaustion in tumor-bearing mouse models and cancer patients [28]. In the experiments testing cytotoxicity of PM21-NK cells against lung cancer spheroids (Figure 3A and Figure 4A–C), TIGIT blockade resulted in the enhancement of cytotoxicity that was increasing as a function of the time in co-culture with tumor, indicating that the rate of killing was decreasing in the absence of TIGIT blockade potentially as a result of progressive decline in function. To determine if TIGIT blockade could alleviate signs of exhaustion in PM21-NK cells upon exposure to tumor spheroids, an in vitro exhaustion model was developed. PM21-NK cells were first co-cultured with A549 spheroids for 7 days either in the presence of anti-TIGIT or isotype control antibodies. NK cells were then stimulated with either PVR^−^ or PVR^+^-K562 cells and the production of effector cytokines and degranulation was assessed. Unexposed, unstimulated PM21-NK cells were used as a negative control while unexposed PM21-NK cells, stimulated with either PVR^+^-K562 or PVR^−^-K562 cells, were used as positive controls. A schematic of the in vitro exhaustion model is shown in Figure 6A, and examples of flow cytometry histograms for one donor are shown in Figure 6B. Notably, the cytotoxicity, cytokine production, and degranulation of unexposed PM21-NK cells stimulated with either PVR^+^-K562 or PVR^−^-K562 cells were comparable and were not affected by TIGIT blockade (Supplementary Figure S8).

When unexposed PM21-NK cells were stimulated with K562 cells, the percentage of NK cells expressing IFNγ increased from less than 1% to 15 ± 5% (*p* = 0.0002) when stimulated with PVR^−^-K562 cells and to 14 ± 7% (*p* < 0.0001) with PVR^+^-K562 cells (Figure 6C). Restimulation with K562 cells after A549 spheroid co-culture resulted in a lower percentage of PM21-NK cells expressing IFNγ (8 ± 5% for PVR^−^ (*p* = 0.03) and 4 ± 3% with PVR^+^ cells (*p* = 0.0002)) (Figure 6C) and the decrease was greater when PVR^+^ cells were used (*p* = 0.04). TIGIT blockade during co-culture with A549 spheroids did not mitigate the tumor-induced decrease in IFNγ expression after restimulation with PVR^−^ cells with still only 9 ± 7% of cells expressing IFNγ. By contrast, restimulation with PVR^+^-K562 cells increased IFNγ expression in NK cells from 3 ± 2% with isotype control to 7 ± 4% with anti-TIGIT, although without reaching significance (*p* = 0.1) (Figure 6C). Additionally, there was no longer a significant difference in IFNγ expression between PVR^−^ or PVR^+^-K562 cell restimulation when anti-TIGIT antibodies were present in the initial tumor co-culture, indicating that the initial difference was TIGIT-dependent. Thus, tumor exposure resulted in close to 80% loss in the IFNγ generation capacity with about 30% of this loss being dependent on TIGIT/PVR engagement.

TNFα generation was also negatively impacted by tumor exposure, with most of the decrease being driven by the TIGIT/PVR axis. Stimulation of unexposed PM21-NK cells with K562 cells resulted in an increased frequency of TNFα^+^ NK cells compared to unstimulated cells (4 ± 1% vs. 29 ± 5% upon PVR^−^ cell stimulation (*p* < 0.0001) and 4% vs. 28 ± 3% with PVR^+^ cells (*p* < 0.0001)) (Figure 6D). Compared to unexposed cells, A549 tumor exposure either in the presence of isotype control or anti-TIGIT antibodies did not result in a change in the frequency of TNFα^+^ NK cells induced in response to PVR^−^-K562 cell restimulation (27 ± 6% in the presence of isotype control and 30 ± 8% with anti-TIGIT antibodies) (Figure 6D). However, restimulation with PVR^+^-K562 cells resulted in a lower frequency of TNFα^+^ NK cells after co-cultures with isotype control antibodies compared to PVR^−^-K562 cell restimulation (27 ± 6% with PVR^−^ vs. 15 ± 5% with PVR^+^ cells; *p* = 0.008) or compared to unexposed NK cells stimulated with PVR^+^-K562 cells (28 ± 3% in unexposed vs. 15 ± 5% for spheroid exposed; *p* = 0.0001) (Figure 6D). Blocking of TIGIT during spheroid co-culture prevented the decrease in the frequency of TNFα^+^ NK cells, with 28 ± 7% of NK cells expressing TNFα after restimulation with PVR^+^-K562 cells, frequencies comparable to unexposed stimulated NK cells (28 ± 3%) and to exposed NK cells stimulated with PVR^−^-K562 cells (27 ± 6%) (Figure 6D). Thus, blocking TIGIT fully restored the TNFα production capacity of NK cells in response to PVR^+^ targets.

TIGIT blockade also restored most of the ability of PM21-NK cells to degranulate upon PVR^+^ cell restimulation post-tumor-co-culture. Surface CD107a expression, a marker for degranulation, increased in unexposed PM21-NK cells upon stimulation with K562 cells where the frequencies of degranulating, CD107a^+^ NK cells increased from 6 ± 3% in unstimulated to 53 ± 9% (*p* < 0.0001) in cells stimulated with PVR^−^K562, and to 44 ± 5% (*p* < 0.0001) after PVR^+^-K562 cell stimulation (Figure 6E). Tumor co-culture in the presence of isotype control antibodies decreased the frequency of CD107a^+^ NK cells upon restimulation with K562 cells (37 ± 5% for PVR^−^ (*p* = 0.004) and 19 ± 8% PVR^+^ (*p* < 0.0001)) with a larger decrease with PVR^+^-K562 cells compared to PVR^−^ cells (*p* = 0.005). TIGIT blockade during tumor co-culture only restored CD107a expression for PVR^+^-K562-restimulated NK cells; resulting in an increase in the frequency of CD107a^+^ NK cells compared to isotype control conditions (41% with TIGIT blockade vs. 19% with isotype control; *p* < 0.0001), resulting in comparable frequencies of CD107a^+^ NK cells to those observed for PVR^−^-K562-restimulated NK cells, which remained unchanged after TIGIT blockade (41% with PVR^+^ cell restimulation vs. 39% with PVR^−^).

In summary, evidence of NK cell exhaustion upon tumor exposure was observed, resulting in decreased frequencies of NK cells degranulating and producing IFNγ and TNFα after re-stimulation with K562 cells compared to K562 stimulation of unexposed NK cells, and these decreases were greater when restimulated with PVR^+^-K562 cells. TIGIT blockade restored the ability of NK cells to produce IFNγ and TNFα and degranulate (based on CD107a surface expression) upon restimulation with PVR^+^ cells in tumor-exposed PM21-NK cells back to levels comparable to re-stimulation with PVR^−^-K562 cells, and for TNFα and degranulation to levels observed for unexposed NK cells stimulated with PVR^+^-K562 cells.

In order to determine if the protective effect of TIGIT blockade on the effector functions of PM21-NK cells post-exposure occurs on a transcriptional level, NK cells were selected after co-culture with A549 spheroids for 7 days and RNA extracted for sequencing and transcriptomic analysis (schematically depicted in Figure 6A). Gene set enrichment analysis revealed that TIGIT blockade upregulated hallmark gene sets including TNFα signaling via NFкB, inflammatory response, IFNγ response, and IFNα response gene sets (Figure 6F, Supplementary Figure S9). These enrichments in the transcriptome indicate a more activated state [70] of PM21-NK cells upon TIGIT blockade during A549 co-culture. Altogether, these observations from functional and transcriptomic analyses demonstrated that TIGIT blockade restored PM21-NK cell anti-tumor functions against PVR^+^ cancer cells after exposure to cancer cell spheroids.

### 3.5. Translational Importance of Activated NK Cells and PVR/TIGIT Axis

**Figure 7:**
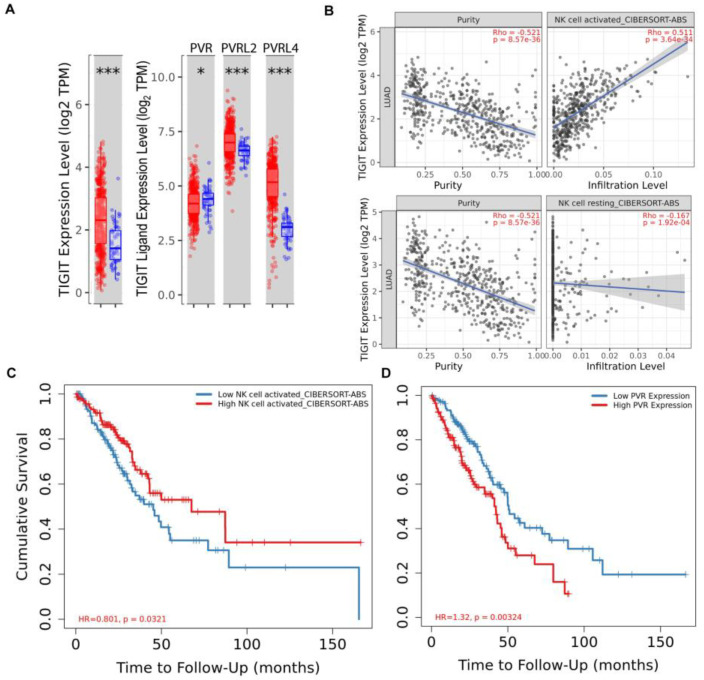
Activated NK cells infiltrated into LUAD tumors upregulate TIGIT and correlate with better survival. The TIMER2.0 webserver was used to analyze the lung adenocarcinoma (LUAD) cohort of The Cancer Genome Atlas (TCGA) (*n* = 515) to determine if there are correlations between NK cell levels, TIGIT/PVR expression, and outcomes. The analysis revealed that TIGIT and its ligands PVRL2 and PVRL4 are significantly upregulated in LUAD tumors compared to normal tissue (**A**). TIGIT expression positively correlated with activated NK cells, but not resting NK cells, based on absolute CIBSORT deconvolution of immune infiltrates and gene expression in the cohort (**B**). Additionally, higher levels of activated NK cell infiltration correlated with better survival (**C**) while higher levels of PVR on tumors correlated with poorer survival (**D**). Statistical significance in (A) was determined by the Wilcoxon test; *p*-values are shown as * if *p* < 0.05 or *** if *p* <0.001). Scatter plots with purity-adjusted spearman’s rho and *p*-value are shown in (**B**). The hazard ratio (HR) and *p*-value for Kaplan-Meier curves are shown in (**C**,**D**).

To assess if there is a correlation between NK cell levels and/or the PVR/TIGIT axis and outcomes, the lung adenocarcinoma (LUAD) cohort of The Cancer Genome Atlas (TCGA) was analyzed using the TIMER2.0 webserver. TIGIT was significantly upregulated in tumor tissue compared to adjacent normal tissue in the LUAD cohort (Figure 7A). While PVR was not upregulated, TIGIT ligands PVRL2 and PVRL4 had significantly increased expression levels (Figure 7A). Further, TIGIT expression positively correlated with infiltrated activated NK cells, but not resting NK cells (Figure 7B), indicating that the presence of activated NK cells in the tissue may lead to higher overall levels of TIGIT expression in tumors, including associated infiltrating immune cells, likely including activated NK cells. Most importantly, higher levels of activated NK cell infiltration correlated to better survival (Figure 7C) while higher levels of PVR on tumors correlated with poorer survival (Figure 7D). There was no correlation between the levels of resting NK cells and outcomes (Supplementary Materials Figure S10). Taken together, these results corroborate the importance of the in vitro findings and show that there is a correlation between levels of activated NK cells, TIGIT tumor tissue expression, and better outcomes. On the other hand, the presence of PVR on tumors would likely result in TIGIT engagement and progression to a more exhausted state correlated with poorer outcomes and also suggest that combining the adoptive transfer of highly activated NK cells with TIGIT blockade could enhance response rates and improve outcomes by preventing exhaustion and prolonging NK-cell activation state.

## 4. Discussion

TIGIT has received a tremendous amount of attention in recent years as a promising target with the potential to match or exceed the success of anti-PD-(L)1 therapies. Based on the exciting preclinical and early clinical data, there are currently over 60 active trials of therapies targeting TIGIT in different cancer settings and in various combinations [71]. There are also more than 20 additional TIGIT-targeting modalities, including antibody–drug conjugates and bi- and tri-specifics, that are currently under development [72]. This high enthusiasm has been recently quenched by the release of data from larger phase III trials in NSCLC (SKYSCRPER-01) and ES-SCLC (SKYSCRAPER-02) that failed to meet primary endpoints in terms of reaching target improvement in progression-free survival or overall survival. Thus, there is a great need to gain a better understanding of TIGIT function in the cell populations that are behind tumor control to realize the potential shortcomings. NK cells are one of those populations that not only kill but also set the stage for other components of the immune system. NK cells also constitutively express TIGIT [13,18,21,22,73,74,75], and, under some circumstances, the levels of TIGIT expression were found to be higher than on any of the T cell subtypes, including CD8 T cells and Tregs [24]. Yet, NK cells are also largely ignored when investigating potential reasons behind the failure of TIGIT therapeutics. This study was designed to provide a more detailed understanding of TIGIT function on activated human NK cells derived from healthy donors prior to, and after, exposure to PVR-expressing lung cancer spheroids. The results were further compared with the analysis of patient data from the LUAD-TCGA database, and together, provide important findings that should be considered when using TIGIT therapeutics as well as adoptive NK cell therapies to potentially improve outcomes.

In the current study, TIGIT was found to be highly expressed on the surface of activated NK cells irrespective of the activation method used. Activation of NK cells with the methods used clinically for the generation of cells for adoptive NK cell therapy, such as cytokines, IL-12/15/18, or through expansion with PM21 particles, resulted in the upregulation of TIGIT expression. TIGIT expression marked NK cells that had increased expression of major activating receptors (CD16, NKG2D, NKp46, and NKp30) and some inhibitory receptors, such as CD96 and TIM-3. TIGIT^+^ NK cells had enhanced cytotoxic function, resulting in a greater level of degranulation and effector cytokine secretion after exposure to tumor targets, suggesting that TIGIT is a marker of NK cell activation. Previous studies have similarly reported that IL-15 stimulation can increase TIGIT expression on NK cells, while other cytokines tested, such as IL-7 and IL-21, resulted in only minor increases (<15%) or no change (such as with IL-12) in the MFI of TIGIT-stained cells [3,25]. In contrast to our findings, the functional performance of TIGIT^+^ NK cells was reported to be lower as compared to TIGIT^−^, although most of the studies examined NK cells derived from tumor-bearing mice or patients either with chronic infection or cancer, which were likely already exhausted [22,26,28,76]. For example, TIGIT expression trended higher in early melanoma but was downregulated in metastatic melanoma [21]. TIGIT^+^ NK cells from melanoma patients were found to have higher lytic potential, as determined by higher levels of perforins and granzyme, but lower lytic function, resulting in lower degranulation and specific lysis. In murine models, TIGIT^+^ NK cells recovered from either spleen or tumors of tumor-bearing mice had a lower frequency of IFNγ-, TNFα-, or DNAM-expressing cells as compared to TIGIT^−^ NK cells [28]. Wang et al. reported that TIGIT^+^ NK cells from healthy individuals had decreased function resulting in lower degranulation and cytokine production, although the stimulation method used was either with IL-12 or lipopolysaccharide (LPS) [24]. Similarly, we did not observe any difference in IFNγ or TNFα production between TIGIT^+^ and TIGIT^−^ NK cells in response to cytokine stimulation, suggesting that these TIGIT^+^ NK cells have greater anti-tumor but not general responsiveness. Thus, the observed differences are likely due to differences in the activation method used for testing the function or the cell source (healthy vs. tumor-derived NK cells). The present study demonstrated that freshly activated and/or expanded NK cells appear to have higher TIGIT expression, which also correlates to a more activated phenotype and enhanced anti-tumor function. Surprisingly, TIGIT blockade also did not affect PM21-NK cell cytotoxicity in short-term assays with 2D lung cancer monolayers. This suggests that activation, likely involving DNAM-1-PVR binding and/or NKG2D, overpowers TIGIT inhibitory signaling in the highly cytotoxic PM21-NK cells [55] in the short term. PM21 NK cells have high expression of major activating receptors such as DNAM-1, NKG2D, NKp46, and NKp30. This also suggests that the observed higher cytotoxicity of expanded cells may also be the result of lower sensitivity to inhibitory signaling.

To address if expanded cells are still susceptible to exhaustion after prolonged tumor exposure, a novel in vitro spheroid model was developed. This model was utilized in combination with blocking antibodies to assess the effect of TIGIT engagement on the function of expanded NK cells after chronic tumor exposure. In this chronic exposure model, TIGIT blockade had no effect within the first 48 h but thereafter gradually improved PM21-NK-cell cytotoxicity against multiple lung cancer spheroids. This suggests that during long-term exposure to tumors, TIGIT signaling can inhibit and/or downregulate NK-cell-mediated killing. Furthermore, chronic tumor exposure had a negative impact on general NK-cell anti-tumor function, resulting in reduced degranulation and decreased expression of effector cytokines IFNγ and TNFα in NK cells exposed to tumors and re-challenged with either PVR^+^ and PVR^−^ K562 cells. Combining TIGIT blockade during tumor exposure with either PVR^+^ or PVR^−^ restimulation allowed us to decern which function was negatively impacted by the engagement of the TIGIT/PVR pathway, and to what extent. PVR/TIGIT interaction was the main mechanism leading to the decline in TNFα production, whereas TIGIT blockade and/or absence of PVR resulted in the retention of full function at the level of unexposed cells. Additional factors contributed to decreased IFNγ production and degranulation in this system, preventing the full restoration of these functions with TIGIT blockade or in the absence of PVR engagement. Although in the present study, the blockade was applied concurrently with tumor exposure and as such likely prevented functional decline, the effect of TIGIT could also be reversing the loss of function, as others have shown when applying anti-TIGIT to cells recovered from tumor microenvironments either from patients or mice [22]. However, in those studies, it is difficult to assess how much of the function is recovered since starting levels are not easy to evaluate. In some of those studies, TIGIT blockade itself was not sufficient to enhance the function of exhausted cells but rather had to be combined with cytokine activation [21,22]. Interestingly, in the present study, no significant changes in the surface phenotype were observed after tumor exposure, including similar DNAM-1 expression, and a trend toward decreased expression was only observed for NKG2D, with some improvement with TIGIT blockade. Transcriptomic analysis of NK cells after exposure to the A549 spheroids demonstrated that chronic TIGIT engagement results in NK cell reprogramming associated with the functional decline, and blocking TIGIT resulted in the upregulation of gene sets involved in inflammatory responses and TNFα signaling, consistent with the functional results. These gene sets could be upregulated by applying TIGIT blockade and suggested a more activated state of NK cells when combined with anti-TIGIT.

These findings agree with previous reports from murine models that showed that in tumor-challenged mice, TIGIT deficiency or blockade resulted in better tumor control and that the recovered tumor-infiltrating NK cells had more than double the frequency of TNFα-producing cells and more CD107^+^ and DNAM^+^ cells within the tumor-infiltrating NK cell populations. There was either small or no change observed in the frequency of IFNγ-producing NK cells dependent on the model, which is somewhat different from the findings presented in this study, although in the current study, the decline observed in IFNγ production appears to be largely mediated by mechanisms other then TIGIT/PVR engagement, where only a minor component was restored with TIGIT blockade and a large decrease was observed with restimulation with PVR^−^ cells. Another difference between the present study and others is in the lack of downregulation of DNAM-1 expression after tumor exposure. Several studies have reported that NK cells obtained from cancer patients or patients with chronic infections had lower expression of DNAM-1 and/or TIGIT [22,77,78]. Moreover, in the tumor-bearing murine models, TIGIT blockade or deficiency resulted in more DNAM-1^+^ cells (~15% more) [28]. This is different from the present study and may be either due to shorter exposure time (7 days vs. >14 days) or due to the greater activation state of the expanded cells that renders the DNAM-1 expression more stable and resistant to downregulation upon TIGIT engagement and/or exhaustion. The cells also retained their function after exposure even in the absence of TIGIT blockade, supporting their utility for adoptive cell therapies.

As mentioned earlier, NK cells are the critical component of anti-tumor immunity. The efficacy of anti-TIGIT antibodies was dependent on NK cells and NK cells were shown to regulate CD8 T cell anti-tumor immunity, including preventing their exhaustion and helping with memory formation [28]. Thus, the presence of functional, TIGIT^+^ NK cells is critical to the efficacy of anti-TIGIT therapies and adoptive NK cell therapy may further improve the efficacy of anti-TIGIT antibodies by boosting overall T cell immune response against cancer cells in cancer patients frequently lacking the NK cell compartment.

NK cells are critically important for the efficacy of anti-TIGIT therapies. Yet, the majority of current therapeutic anti-TIGIT antibodies in development, including tiragolumab that recently failed in PhIII trials, are Fc-competent and can engage FcγR on effector cells, e.g., macrophages, and potentially lead to clearance of TIGIT^+^ cells. Depletion of TIGIT^+^ populations including Tregs has been reported in tumor samples obtained from patients treated with Fc-optimized TIGIT antibodies and was considered important for the therapeutic effects of the antibodies [79,80]. Given that TIGIT is highly expressed on activated, tumor-infiltrating NK cells and at lower levels on resting non-activated NK cells (Figure 1 and Figure 7), Fc-competent anti-TIGIT antibodies could lead to the depletion of this critical population through FcR-dependent mechanisms. The effect of an Fc-competent anti-TIGIT antibody on the function of NK cells has been examined in a short-term assay and shown to increase the percentage of CD107a^+^ NK cells [81]; however, long-term assays or in-depth analyses of the effects on NK cell amount were not performed. Although blocking TIGIT could help prevent NK cell exhaustion and/or restore NK cell function, Fc-competent anti-TIGIT antibodies may negatively affect the efficacy of treatment by removal of this critical effector population. Further studies are warranted to probe this potential consequence of Fc-competent antibody use and its effect on treatment outcomes.

## 5. Conclusions

**Figure 8:**
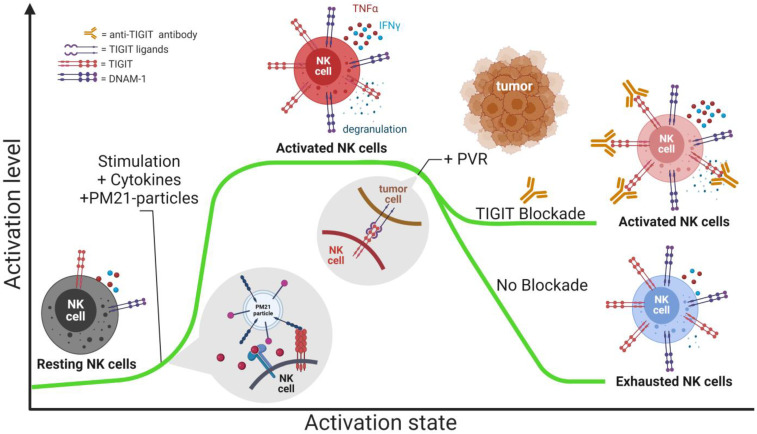
TIGIT expression in the context of NK cell activation state and TIGIT blockade. TIGIT expression is increased on activated NK cells compared to resting. Activated TIGIT^+^ NK cells have a better anti-tumor response as compared to TIGIT^−^ NK cells. However, chronic TIGIT engagement with its ligands in the tumor microenvironment leads to the functional decline of NK cells, which can be prevented with anti-TIGIT.

In summary, this study provides insight into mechanisms of TIGIT blockade in ex vivo-expanded human NK cells. TIGIT is highly expressed on activated NK cells and correlates with higher anti-tumor function, although during prolonged tumor exposure, its engagement can result in a decrease in NK cell function (Figure 8). Anti-TIGIT antibodies have the potential to improve the efficacy of PM21-NK cell tumor immunity, and adoptive PM21-NK cells and anti-TIGIT antibodies should be explored in future preclinical studies and clinical trials as a combination therapy against lung tumors.

## Figures and Tables

**Table 1:** TIGIT ligand expression in lung cancer cell lines. Lung cancer cell lines were stained with TIGIT-ligand-specific antibodies and compared to isotype controls to determine ligand expression by flow cytometry. Data are presented as percentage of cancer cells that are ligand-positive (%) averaged from 2–3 different passages, in duplicate.

Lung Cancer Cell Line	PVR (%)	PVRL2 (%)	PVRL3 (%)	PVRL4 (%)
A549	100 ± 1	100 ± 1	2 ± 1	1 ± 1
NCI-H358	100 ± 1	100 ± 1	0 ± 1	82 ± 18
NCI-H1299	100 ± 1	100 ± 1	2 ± 1	1 ± 1
NCI-H1975	100 ± 1	100 ± 1	2 ± 2	16 ± 13

## Data Availability

Raw RNA sequencing data are deposited in the NCBI SRA database under submission number SUB12437119 and data will be made public and accession numbers provided prior to publication.

## Supplementary Materials

The following supporting information can be downloaded at: https://www.mdpi.com/article/10.3390/cancers15102712/s1, Supplemental Methods, Figure S1: Example gating strategy for counting viable NK by flow cytometry; Figure S2: PM21-NK cell expansion; Figure S3: Cytokine-preactivated TIGIT^+^ NK cells have increased TNFα and degranulation in response to K562 cells compared to TIGIT^−^ cytokine-preactivated NK cells; Figure S4: TIGIT^+^ PM21-NK cells have the same response as TIGIT^−^ PM21-NK cells when exposed to cytokine stimulation; Figure S5: TIGIT ligand expression on cancer cells, Figure S6: Kinetic live-cell imaging cytotoxicity assay; Figure S7: PM21-NK cell phenotype does not change after tumor exposure in the presence of anti-TIGIT; Figure S8: Short-term TIGIT blockade does not further enhance PM21-NK cell function; Figure S9: Supplemental RNA-seq data; Figure S10: LUAD patient outcomes stratified by resting NK cell infiltration.
