# Supplementary material for: TIGIT Expression on Activated NK Cells Correlates with Greater Anti-Tumor Activity but Promotes Functional Decline upon Lung Cancer Exposure: Implications for Adoptive Cell Therapy and TIGIT-Targeted Therapies
Source: Cancers (Basel). 2023 May 11;15(10):2712. doi: 10.3390/cancers15102712 (PMC10216728; doi:10.3390/cancers15102712)
Supplement: Supplementary file 1 [file cancers-15-02712-s001.zip › cancers-2333884-supplementary.pdf]

Supplemental Methods

Annexin V cytotoxicity assay

PM21-NK cells were co-cultured with target PVR^+ or PVR^- K562-GFPLuc cells at indicated effector *vs.* target (E:T) ratios in the presence of Ultra-LEAF isotype or anti-TIGIT antibodies (Biolegend, San Diego, CA, USA) for 60 minutes at 37°C in a tissue culture incubator. Cells were then centrifuged and stained with an Annexin-V-Pacific Blue antibody, incubated for 15 minutes at 4°C and analyzed by flow cytometry. The cytotoxicity was determined based on the absolute amount of Viable Target Cells (GFP^+/Annexin V^-) remaining in each well with effectors ($VTC^{E:T}$) and referenced to average VTC in "target alone" control wells ($VTC^{T\ ctrl}$)

$$\boldsymbol{Cytotoxicity^{E:T}}(\%) = \left(\mathbf{1} - \frac{\boldsymbol{VTC^{E:T}}}{\boldsymbol{Avg\ VTC^{T ctrl}}}\right) \times \mathbf{100}$$

IFNγ and TNFα expression, and degranulation

30,000 NK cells were co-cultured with PVR^- or PVR^+ K562 cells in the presence of Ultra-LEAF isotype or anti-TIGIT antibodies (Biolegend, San Diego, CA, USA) for 4-6 hours in the presence of Brefeldin A and Golgi Stop™ at 37°C. Samples were stained with extracellular target protein-specific antibodies (CD3, CD56 and CD107a). NK cells were then fixed and permeabilized (eBiosciences IC Fixation and permeabilization buffers) and stained for intracellular protein targets (IFNγ and TNFα). Data was acquired by flow cytometry and analyzed by FlowJo software.

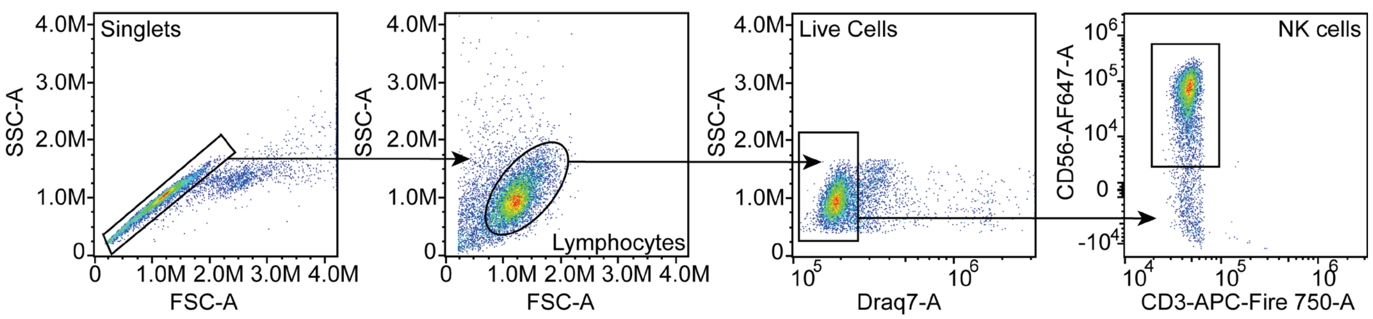

Supplemental Figure S1. Example gating strategy for counting viable NK cells by flow cytometry. Using flow cytometer software, an FSC (height) vs FSC (area) dot plot was prepared and gated on the single cell population ("singlets"). From the "singlets" population, an FSC-A vs SSC-A dot plot was prepared and gated on "lymphocytes". From the "Lymphocytes" populations, a viability gate was applied using an SSC-A vs DRAQ7™ dot plot, to gate on DRAQ7™ negative "live cells" for some experiments. Finally, a CD56 vs CD3 dot plot was prepared, gating on the CD56 $^{+}$/CD3^{-} population for "NK cells". An example gating strategy is shown.

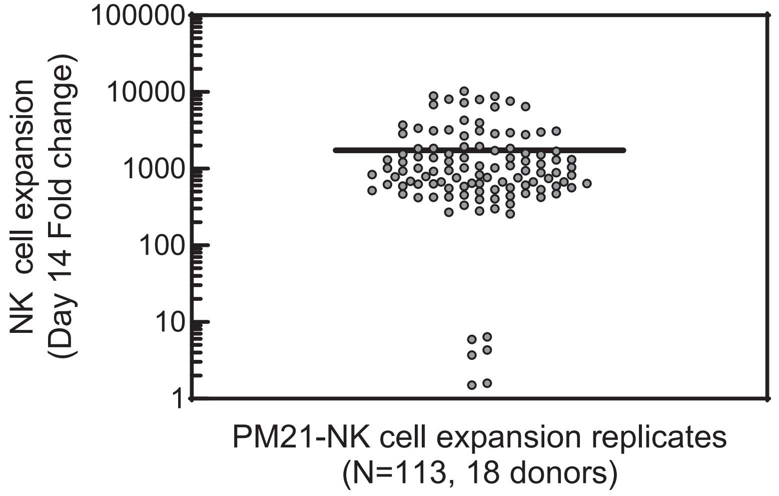

Supplemental Figure S2. **PM21-NK cell expansions**. Peripheral blood mononuclear cells were separated by density gradient from buffy coats of de-identified healthy donors. T cell-depleted PBMCs were stimulated with PM21-particles and cultured for 14 days to be used for various experiments. This figure represents the cumulative results of PM21-NK cell expansions conducted over one year period (N=113 from 18 donors). Based on these data, the method results in an average 1,741-fold, 95% CI [1,341 to 2,141-fold], expansion in 14 days.

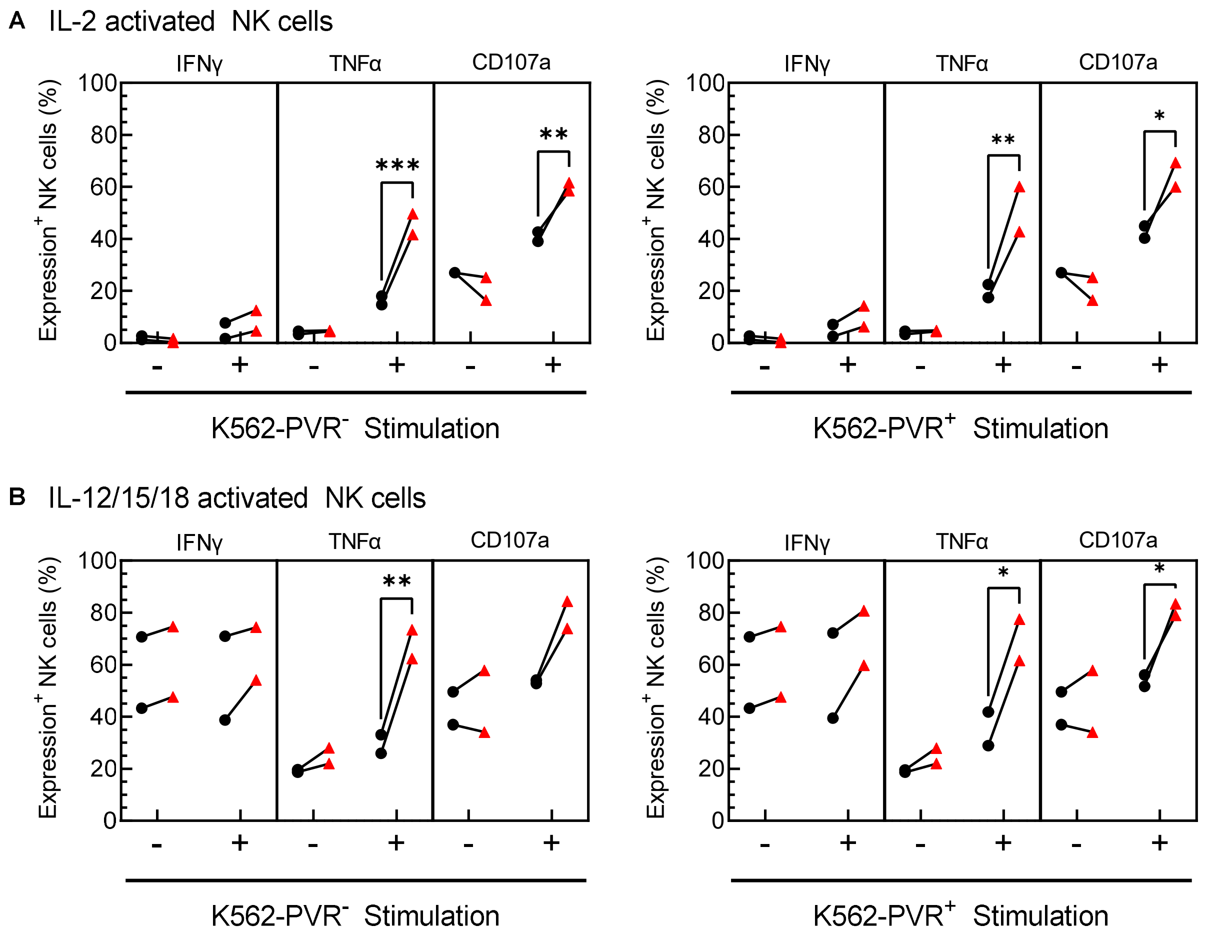

Supplemental Figure S3. **Cytokine-preactivated TIGIT^{+} NK cells have increased TNFα and degranulation in response to K562 cells compared to TIGIT^{-} cytokine-preactivated NK cells.** NK cells were isolated from thawed PBMCs and activated overnight with either IL-2 (**A**) or IL12/15/18 (**B**). Cytokine-activated NK cells were then stimulated with either PVR^{-}-K562 or PVR^{+}-K562 cells for 4 hours. The percent of TIGIT^{-} NK cells (black circles) and TIGIT^{+} NK cells (red triangles) expressing IFNγ, TNFα and degranulation marker CD107a were determined (N=2 donors in duplicate). Significantly more TIGIT^{+} NK cells produced TNFα in response to both PVR^{-} and PVR^{+} K562 cells compared to TIGIT^{-} NK cells preactivated with IL2 ($p=0.0007$ for PVR^{-} and $p=0.0079$ for PVR^{+} K562 stimulation) or preactivated with IL12/15/18 ($p=0.0017$ for PVR^{-} and $p=0.01$ for PVR^{+} K562 stimulation). Similarly, more TIGIT^{+} NK cells expressed more CD107a in response to both PVR^{-} and PVR^{+} K562 cells compared to TIGIT^{-} NK cells preactivated with IL2 ($p=0.0055$ for PVR^{-} and $p=0.01$ for PVR^{+} K562 stimulation) or preactivated with IL12/15/18 ($p=0.065$ for PVR^{-} and $p=0.049$ for PVR^{+} K562 stimulation). No significant difference in IFNγ production was observed.

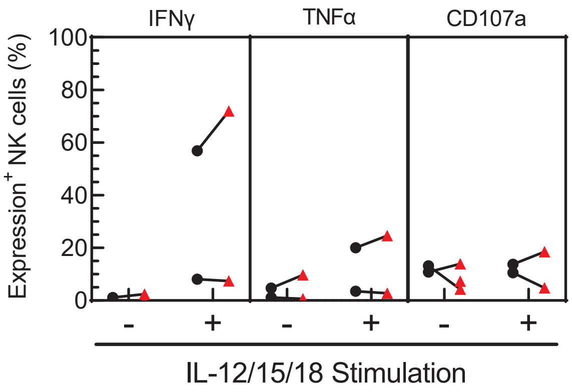

Supplemental Figure S4. **TIGIT^{+} PM21-NK cells have a comparable response as TIGIT^{-} PM21-NK cells when stimulated with cytokines.** PM21-NK cells were either unstimulated or stimulated with IL12/IL15/IL18 cytokines for 4 hours. The percent of TIGIT^{-} PM21-NK cells (black circles) and TIGIT^{+} PM21-NK cells (red triangles) expressing IFNγ, TNFα, and degranulation marker CD107a were determined (N=2 donors in duplicate). No difference in the percentage of NK cells expressing IFNγ, TNFα or degranulation marker CD107a was observed between TIGIT^{+} and TIGIT^{-} PM21-NK cells.

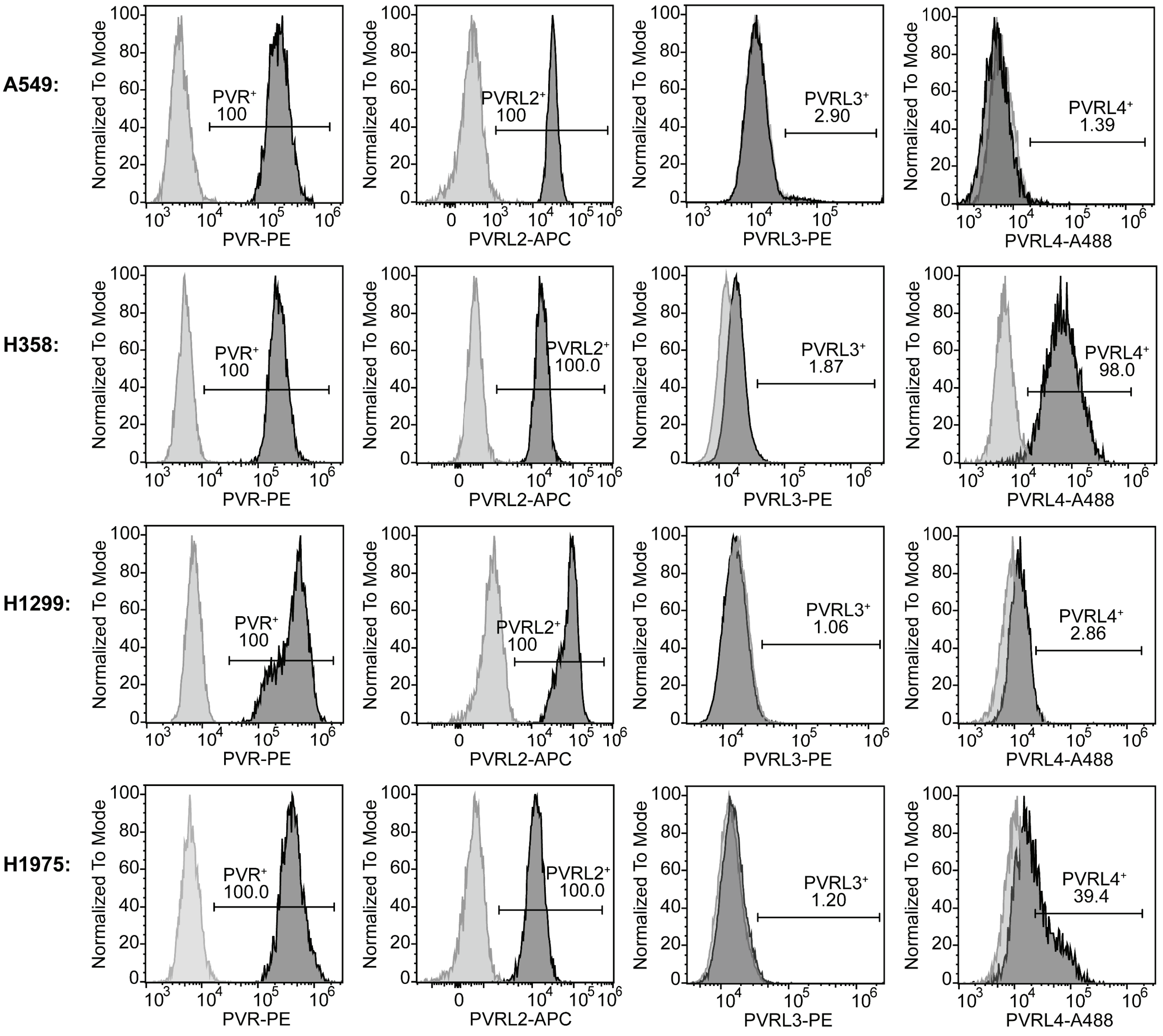

Supplemental Figure S5. TIGIT ligand expression on cancer cells. Lung tumor cell lines used in this study were analyzed for the presence of PVR, PVRL2, PVRL3, and PVRL4 expression by flow cytometry. Representative histogram overlays are shown with isotype control in light gray and ligand-specific staining in dark gray. The percent of cells positive for each ligand is shown above the gate.

A

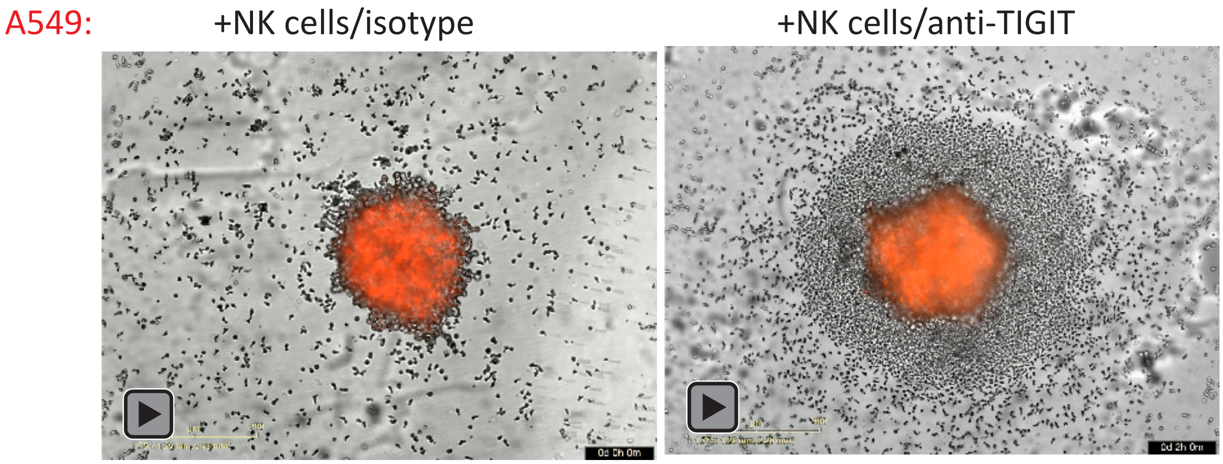

B

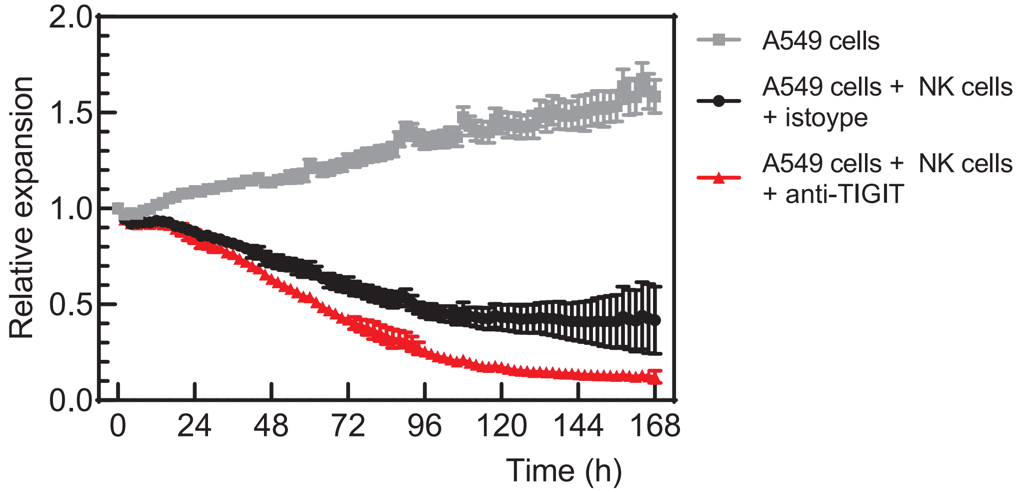

Supplemental Figure S6. Kinetic live-cell imaging cytotoxicity assay. Representative videos of live-cell imaging cytotoxicity assays with A549-NLR spheroids (red) and 10,000 NK cells added in the presence of isotype (left video) or anti-TIGIT antibodies (right video)(**A**). Representative raw data from one donor in triplicate is shown for A549 relative expansion alone (gray squares) or the presence of 0.3:1 NK cells:A549 cells with isotype control (black circles) or anti-TIGIT (red triangles) **(B).**

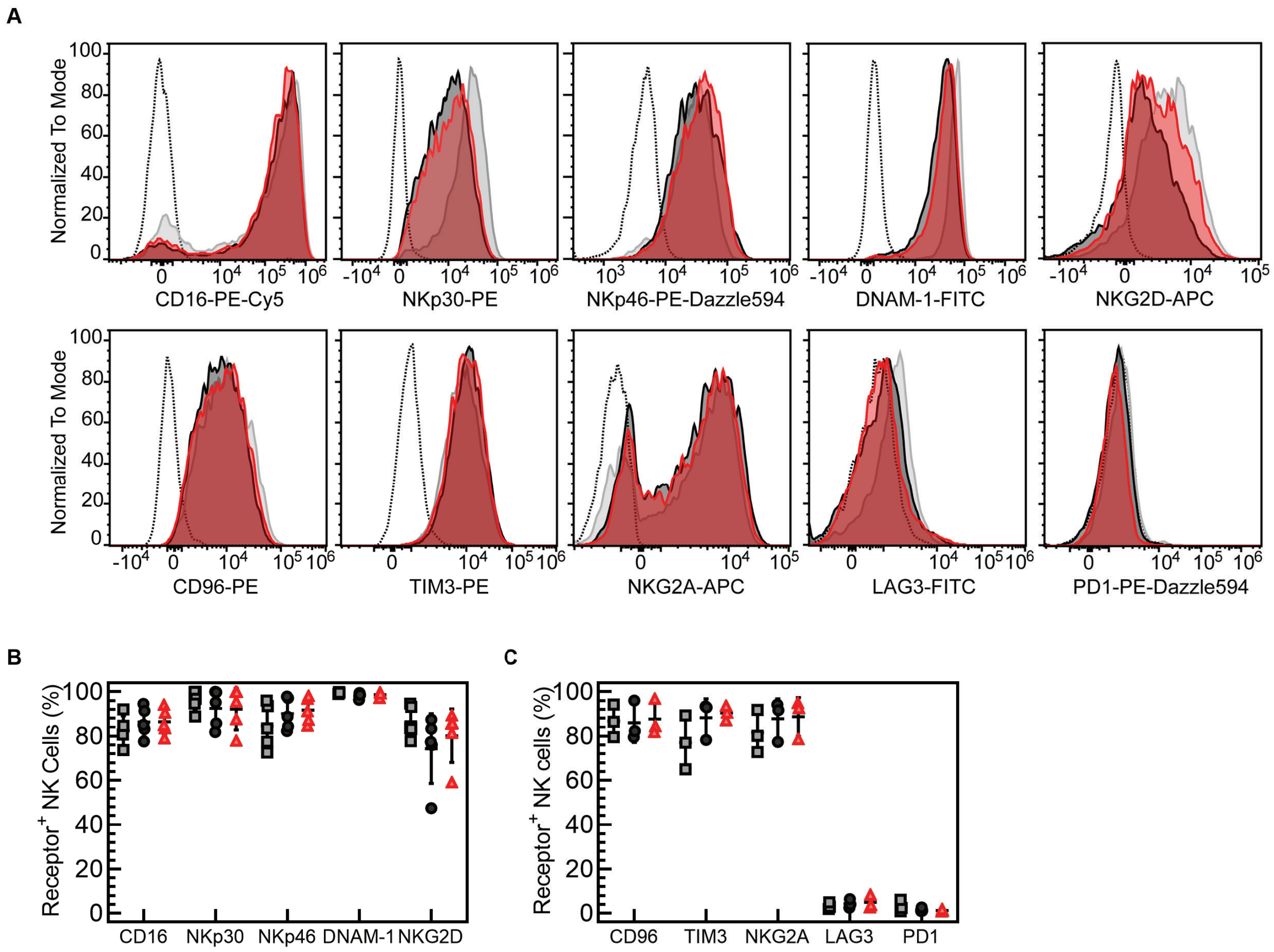

Supplemental Figure S7. PM21-NK cell phenotype does not change after tumor exposure in the presence of anti-TIGIT. After 7 days of co-culture, NK cells were analyzed to determine inhibitory and activating receptors' expression by flow cytometry. Representative flow cytometry histograms are shown overlaying isotype control (dotted line), unexposed PM21-NK cells (gray outline, light gray fill) and PM21-NK cells exposed to A549 spheroids in the presence of isotype control (black outline, dark gray fill) or anti-TIGIT (red outline, red fill). (**A**).TIGIT blockade did not significantly change the expression of any major inhibitory (**B**) or activating receptors (**C**) when co-cultured with A549 spheroids in the presence of anti-TIGIT (red triangles) compared to isotype control (black circles) and expression levels were similar to unexposed NK cells (gray squares) (N=3-5 donors in duplicate).

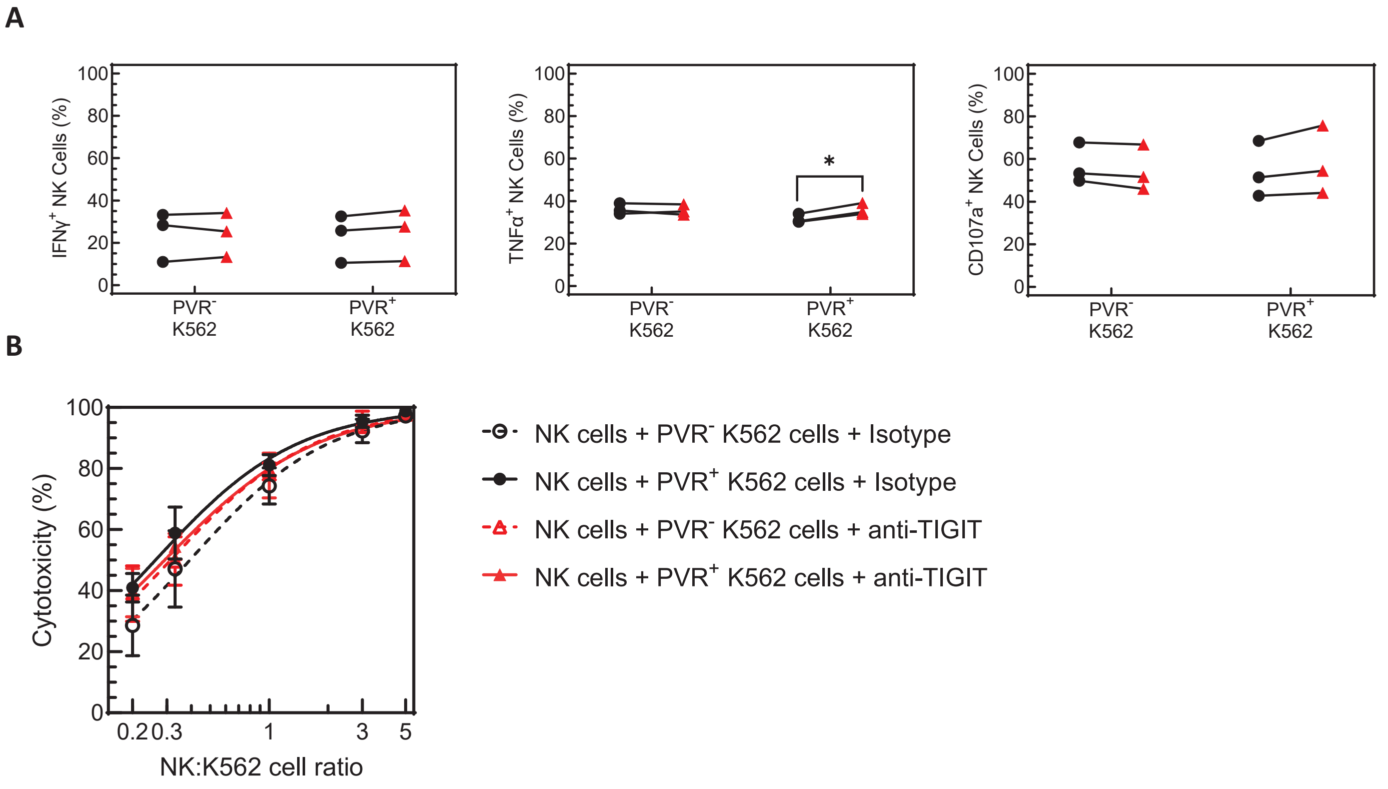

Supplemental Figure S8. Short-term TIGIT blockade does not further enhance PM21-NK cell function. NK cells were expanded with PM21-particles from T cell-depleted PBMCs obtained from multiple donors. These expanded NK cells were co-cultured with K562 cancer cells with or without PVR expression for 4-6 hours in the presence of anti-TIGIT or isotype control with Brefeldin A and Golgi Stop. Expression of IFNγ and TNFα and surface CD107a was analyzed by flow cytometry. Compared to NK cells alone (black circles) TIGIT blockade (red triangles) did not significantly change the percentage of donor-matched NK cells expressing IFNγ **(A, left panel),** TNFα **(A, middle panel**), or surface CD107a **(A, right panel**) upon stimulation with PVR$^-$ K562 cells or PVR$^+$ K562 cells, except a small significant increase in TNFα expressing NK cells upon TIGIT blockade (36% ± 3% *vs.* 32% ± 2% p = 0.008) (N=3 donors in duplicate). The effect of short-term TIGIT blockade on PM21-NK cell cytotoxicity was also determined. PM21-NK cells were co-cultured with PVR$^-$ or PVR$^+$ K562 cells in the presence of anti-TIGIT or isotype control antibodies for 1 hour and NK cell cytotoxicity was determined by annexin V staining of the K562 cells (N=2 donors in duplicate). Concentration-dependent cytotoxicity curves were generated using multiple NK:K562 ratios and the area under the curve determined. Short-term TIGIT blockade did not significantly improve PM21-NK cell cytotoxicity against either PVR$^-$ or PVR$^+$ K562 cells compared to isotype control antibodies (335 ± 6 % · ratio *vs* 325 ± 10 % · ratio against PVR$^-$ K562 cells and 346 ± 3 % · ratio *vs* 341 ± 10 % · ratio against PVR$^+$ K562 cells) (**B**).

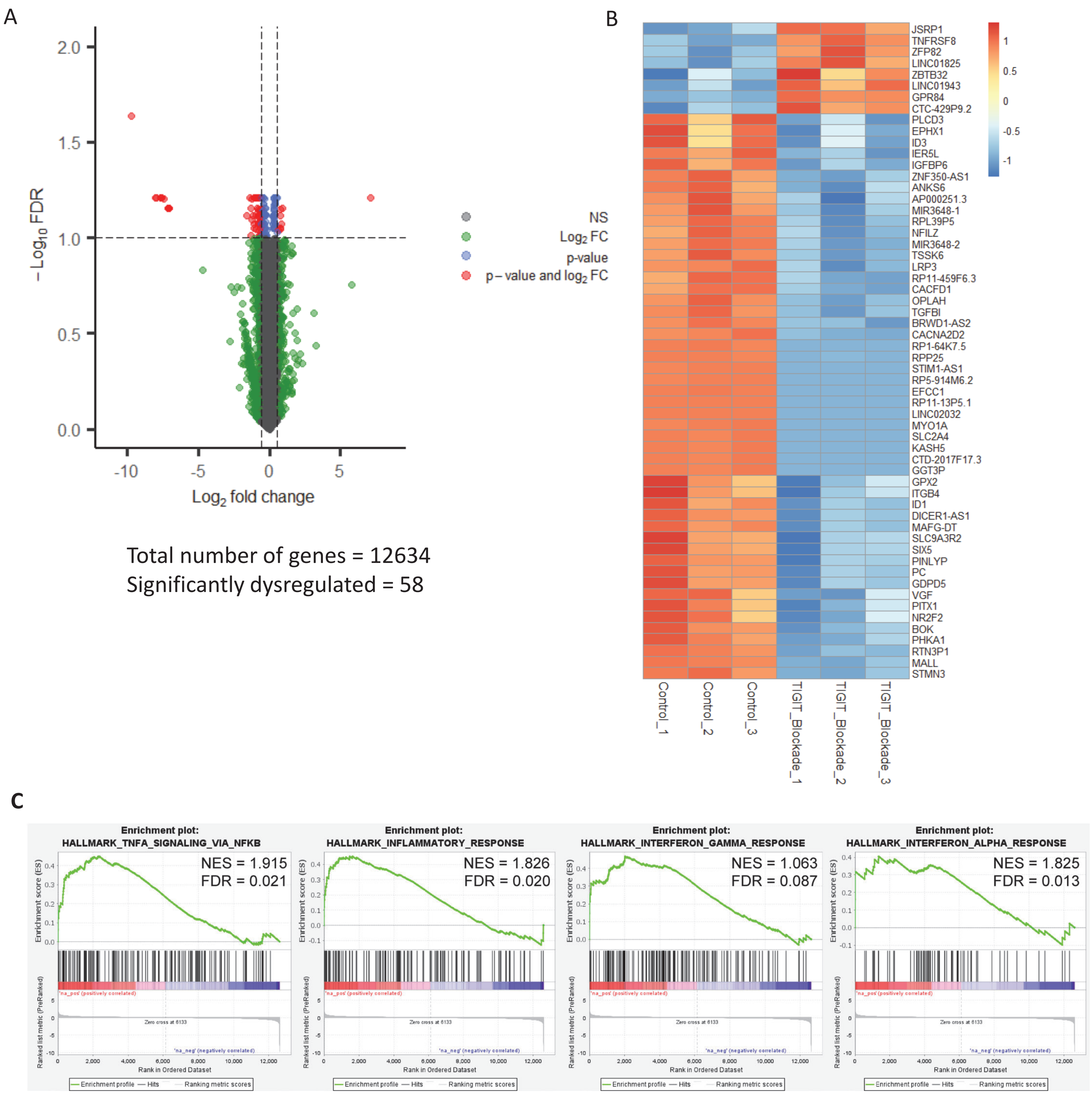

Supplemental Figure S9. Supplemental RNA-seq data. Volcano plot (**A**) and heatmap (**B**) for significantly differentially expressed genes with FDR < 0.1 and -0.58 > Log2 fold change > 0.58 are shown based on RNA-seq data comparing NK cells from 3 donors exposed to tumor in the presence of isotype control or anti-TIGIT. Gene set enrichment (GSEA) analysis of RNA-seq data shows TIGIT blockade upregulated IFNγ, TNFα, and other related inflammation response gene sets. Enrichment plots for top upregulated gene sets are presented with their corresponding NES and FDR value (**C**).

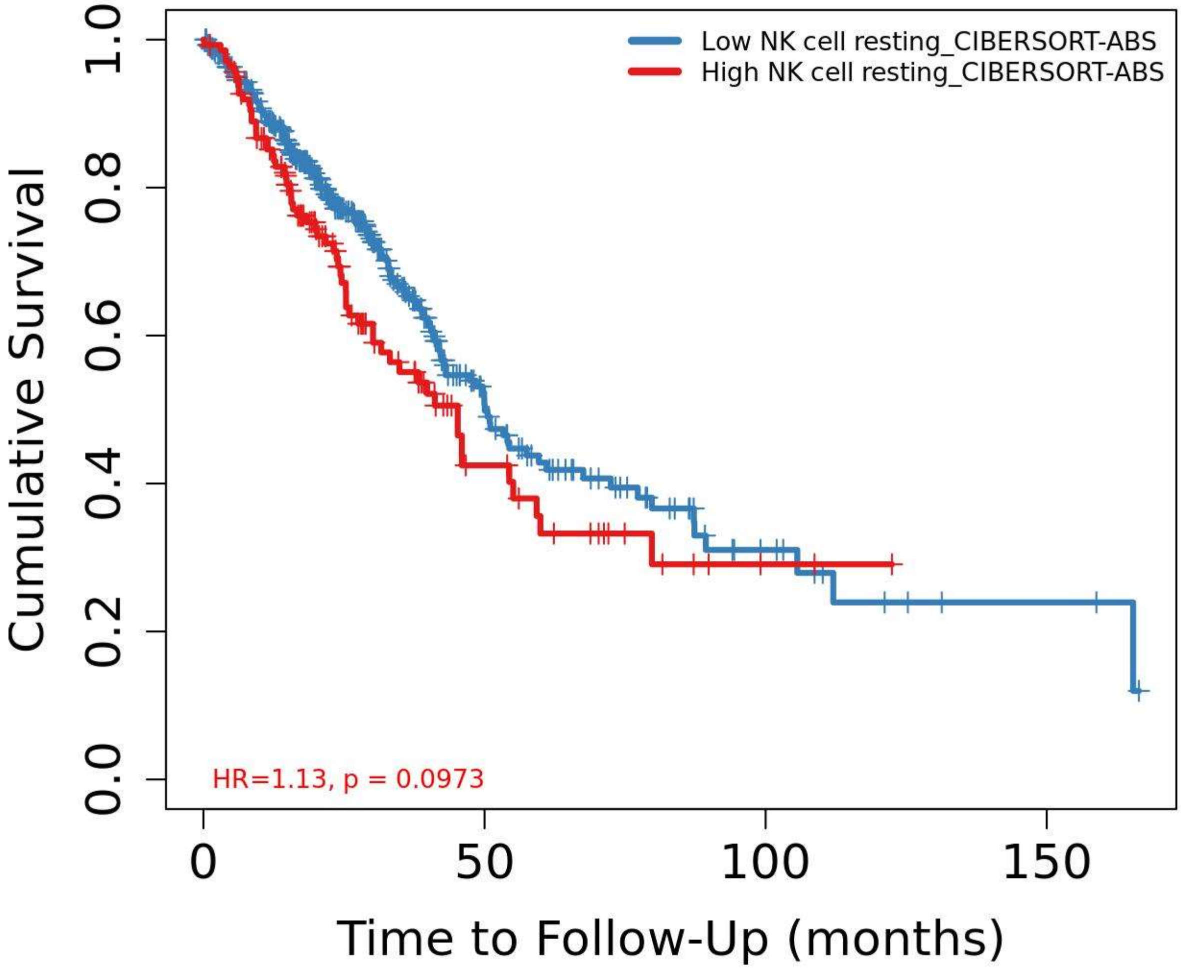

Supplemental Figure S10. LUAD patient outcomes stratified by resting NK cell infiltration. LUAD-TCGA patient cohort survival correlation comparing cumulative survival of low resting NK cell resting infiltration (blue) and high resting NK cell infiltration (red) using a 50% cutoff between patient populations. Hazard ratio (HR) and *p*-value for Kaplan-Meier curves are shown.
